# Systematic Prioritization of Candidate Genes in Disease Loci Identifies *TRAFD1* as a Master Regulator of IFNγ Signaling in Celiac Disease

**DOI:** 10.3389/fgene.2020.562434

**Published:** 2021-01-25

**Authors:** Adriaan van der Graaf, Maria M. Zorro, Annique Claringbould, Urmo Võsa, Raúl Aguirre-Gamboa, Chan Li, Joram Mooiweer, Isis Ricaño-Ponce, Zuzanna Borek, Frits Koning, Yvonne Kooy-Winkelaar, Ludvig M. Sollid, Shuo-Wang Qiao, Vinod Kumar, Yang Li, Lude Franke, Sebo Withoff, Cisca Wijmenga, Serena Sanna, Iris Jonkers, BIOS Consortium

**Affiliations:** ^1^Department of Genetics, University Medical Center Groningen, University of Groningen, Groningen, Netherlands; ^2^Estonian Genome Center, Institute of Genomics, University of Tartu, Tartu, Estonia; ^3^Department of Immunology, K. G. Jebsen Coeliac Disease Research Centre, University of Oslo, Oslo, Norway; ^4^Deutsches Rheumaforschungszentrum Berlin (DRFZ), An Institute of the Leibniz Association, Berlin, Germany; ^5^Charité–Universitätsmedizin Berlin, Corporate Member of Freie Universität Berlin, Humboldt-Universität zu Berlin, and Berlin Institute of Health, Department of Gastroenterology, Infectious Diseases and Rheumatology, Berlin, Germany; ^6^Department of Immunology, Leiden University, Leiden, Netherlands; ^7^Department of Internal Medicine and Radboud Center for Infectious Diseases (RCI), Radboud University Medical Center, Nijmegen, Netherlands; ^8^Department of Computational Biology for Individualised Infection Medicine, Centre for Individualised Infection Medicine, Helmholtz Centre for Infection Research, Hannover Medical School, Hanover, Germany; ^9^Istituto di Ricerca Genetica e Biomedica (IRGB) del Consiglio Nazionale delle Ricerche (CNR), Monserrato, Italy

**Keywords:** celiac disease, gene prioritization, expression quantitative trait locus (eQTL), *TRAFD1*, trans regulation

## Abstract

Celiac disease (CeD) is a complex T cell-mediated enteropathy induced by gluten. Although genome-wide association studies have identified numerous genomic regions associated with CeD, it is difficult to accurately pinpoint which genes in these loci are most likely to cause CeD. We used four different *in silico* approaches—Mendelian randomization inverse variance weighting, COLOC, LD overlap, and DEPICT—to integrate information gathered from a large transcriptomics dataset. This identified 118 prioritized genes across 50 CeD-associated regions. Co-expression and pathway analysis of these genes indicated an association with adaptive and innate cytokine signaling and T cell activation pathways. Fifty-one of these genes are targets of known drug compounds or likely druggable genes, suggesting that our methods can be used to pinpoint potential therapeutic targets. In addition, we detected 172 gene combinations that were affected by our CeD-prioritized genes in *trans*. Notably, 41 of these *trans*-mediated genes appear to be under control of one master regulator, *TRAF-type zinc finger domain containing 1* (*TRAFD1*), and were found to be involved in interferon (IFN)γ signaling and MHC I antigen processing/presentation. Finally, we performed *in vitro* experiments in a human monocytic cell line that validated the role of *TRAFD1* as an immune regulator acting in *trans*. Our strategy confirmed the role of adaptive immunity in CeD and revealed a genetic link between CeD and IFNγ signaling as well as with MHC I antigen processing, both major players of immune activation and CeD pathogenesis.

## Introduction

Celiac disease (CeD) is an autoimmune disease in which patients experience severe intestinal inflammation upon ingestion of gluten peptides. CeD has a large genetic component, with heritability estimated to be ~75% (Kuja-Halkola et al., 2016). The largest CeD-impacting locus is the human leukocyte antigen (HLA) region, which contributes ~40% of CeD heritability (Bevan et al., 1999). While the individual impacts of CeD-associated genes outside the HLA region are smaller, they jointly account for an additional 20% of heritability. Previous genome-wide association studies (GWASs) have identified 42 non-HLA genomic loci associated with CeD. Yet, identification of these non-HLA genetic components and an understanding of the molecular perturbations associated with them are necessary to understand CeD pathophysiology.

Understanding the biological mechanisms of non-HLA CeD loci is difficult: only three of these loci point to single-nucleotide polymorphisms (SNPs) located in protein-coding regions (Trynka et al., 2011). The other CeD-risk loci cannot be explained by missense mutations, making it necessary to look at other biological mechanisms such as gene expression to explain their role in CeD pathogenicity. Several studies have been performed to integrate expression quantitative trait loci (eQTLs) with CeD GWAS associations (Dubois et al., 2010; Kumar et al., 2015; Ricaño-Ponce et al., 2016), and several candidate genes, including *UBASH3A, CD274, SH2B3*, and *STAT4* (Zhernakova et al., 2011), have been identified, implicating T cell receptor, nuclear factor (NF)κB, and interferon (IFN) signaling pathways as biological pathways associated with CeD pathology. Unfortunately, these eQTL studies had limited sample sizes, which reduced their power to identify *cis*- and (especially) *trans*-eQTLs. Furthermore, previous attempts to integrate eQTLs have mostly annotated genomic loci based on cataloged eQTLs without testing the causality of the genes in the onset or exacerbation of CeD (Ricaño-Ponce et al., 2016; Jonkers and Wijmenga, 2017; Fernandez-Jimenez and Bilbao, 2019).

Gene expression and GWAS data can also be integrated using methodologies that identify shared mechanisms between diseases. These methods can be roughly divided into three classes: variant colocalization methods, causal inference methods, and co-expression methods. Colocalization methods consider the GWAS and eQTL summary statistics at a locus jointly and probabilistically test if the two signals are likely to be generated by the same causal variant (Giambartolomei et al., 2014). Causal inference methods test if there is a causal relationship between expression changes and the disease using genetic associations to remove any confounders (Burgess et al., 2013; van der Graaf et al., 2020). Finally, co-expression methods do not use eQTL information, but rather test if there is significant co-expression between the genes that surround the GWAS locus (Pers et al., 2015). Unfortunately, there is no current “gold standard” method for finding the causal gene behind a GWAS hit, as all the methods discussed here are subject to their respective assumptions, drawbacks, and caveats. However, it is worthwhile to use all these methods in parallel to find the most likely causal genes for CeD.

Here, we systematically applied four prioritization methods to the latest meta-analysis for CeD that we have performed previously (Ricaño-Ponce et al., 2020) and coupled them with eQTL results from the Biobank Integrative Omics Study (BIOS) cohort (Zhernakova et al., 2017), one of the largest cohorts for which there is genotype and RNA sequencing (RNA-seq) expression data of peripheral blood mononuclear cells. We focused on 58 CeD-associated loci (*p* < 5 × 10^−6^) outside the HLA region. Our approach prioritized 118 genes in 50 loci and identified one gene, *TRAF-type zinc finger domain containing 1* (*TRAFD1*), as a master regulator of *trans*-effects. We then experimentally validated the role of *TRAFD1*-mediated genes using a *TRAFD1* knockdown through RNA-seq in a disease-relevant cell type. Our study yields novel insights into the genetics of CeD and is proof-of-concept for a systematic approach that can be applied to other complex diseases. A schematic overview of our study is shown in Supplementary Figure 1A.

## Nomenclature

Underlined words are definitions that have been explained in the preceding lines.

**eQTL**, expression quantitative trait locus, a location on the genome that is statistically associated with changes in gene expression.

***cis*-eQTL**, an eQTL located in the same locus of the gene that is being interrogated (within 1.5 Mb from gene transcript start or end).

***trans*-eQTL**, an eQTL that is not physically close to the gene that is being interrogated (>1.5 Mb from transcript start/end or on a different chromosome).

***cis*-eQTL gene**, a gene that is associated with a change in expression as a consequence of a *cis*-eQTL.

***trans*-eQTL gene**, a gene that is associated with a change in expression as a consequence of a *trans*-eQTL.

**CeD**, celiac disease

**CeD-associated region**, a genomic region that is associated with CeD based on results from genome-wide association studies on CeD.

**Prioritized gene**, a gene prioritized as being potentially causal for CeD according to the four statistical methods depicted in Supplementary Figures 1B,C. In this study, prioritized genes are always within the CeD-associated regions.

**Mediating** ***cis* gene**, a prioritized gene that is statistically responsible for the change in expression of a *trans*-eQTL gene. Of note, while the *trans*-eQTL is located in the same CeD-associated region of the mediating *cis*-gene, the mediated *trans-*gene is not.

**Mediated trans gene**, a gene located outside CeD-associated regions that is statistically mediated by a mediating cis gene located in the same region of the corresponding *trans*-eQTL.

## Materials and Methods

### Genotypes for Expression Quantitative Trait Loci Analysis

We used the BIOS cohort (Zhernakova et al., 2017) to map eQTLs in 3,746 individuals of European ancestry. The BIOS cohort is a collection of six cohorts: the Cohort on Diabetes and Atherosclerosis Maastricht (van Greevenbroek et al., 2011), the Leiden Longevity Study (Deelen et al., 2016), Lifelines DEEP (Tigchelaar et al., 2015), the Netherlands Twin Registry (Lin et al., 2016), the Prospective ALS Study Netherlands (Huisman et al., 2011), and the Rotterdam Study (Hofman et al., 2015). As described in Võsa et al. (2018), each cohort was genotyped separately using different arrays, and genotypes were subsequently imputed to the Haplotype Reference Consortium panel (HRC v1.0) on the Michigan imputation server (Das et al., 2016).

While genotyping data were generated and imputed independently of this study (Võsa et al., 2018), here we applied additional filters at the marker and sample level. Specifically, we considered only biallelic SNPs with a minor allele frequency (MAF) >0.01, a Hardy–Weinberg test *p* > 10^−6^, and an imputation quality RSQR > 0.8. We also removed related individuals using a genetic relationship matrix (GRM) created with PLINK 1.9 (Chang et al., 2015). Pairs of individuals with a GRM value >0.1 were considered related, and one individual was removed from each of these pairs. Furthermore, population outliers were identified using a principal component analysis on the GRM, and individuals who were more than 3 standard deviations from the means of principal component 1 or 2 were removed.

### Expression Quantification and Expression Quantitative Trait Loci Analysis

We used preprocessed RNA-seq data from a previous study (Zhernakova et al., 2017; Võsa et al., 2018). After matching the RNA-seq data with the genotyping data filtered described above, 3,503 individuals, 19,960 transcripts, and 7,838,327 autosomal SNPs remained for analyses. We performed genome-wide eQTL mapping for the transcripts using PLINK 1.9 (Chang et al., 2015) with the –assoc command. We defined *cis*-eQTL variants as those located within ± 1.5 Mb of the transcript and *trans*-eQTLs as variants located outside these boundaries. Of note, we opted for this larger window for defining *cis*-eQTLs (usually is ± 500 Kb) so that eQTL associations would fully overlap the associated CeD GWAS peak even when a gene is on the edge of the CeD-associated region (chosen to be 1 Mb). To select variants that could explain the *cis*-eQTL signal of a gene, we used GCTA-COJO (Yang et al., 2011) v1.26. For this analysis, we required selected variants to reach a *p*-value threshold of 5 × 10^−6^ and included the BIOS cohort genotypes as LD reference. This identified 707 genes with at least one eQTL reaching this threshold, 357 of which had more than one conditionally independent eQTL variant.

### Celiac Disease Summary Statistics Associated Regions and Candidate Genes

We used summary statistics from a CeD GWAS meta-analysis of 12,948 cases and 14,826 controls that analyzed 127,855 variants identified using the ImmunoChip array (Ricaño-Ponce et al., 2020). SNP positions were lifted over to human genome build 37 using the UCSC *liftover* tool. We first identified lead associated variants in the CeD meta-analysis by performing *p*-value clumping: we used PLINK 1.9 (Chang et al., 2015) to select variants at a *p*-value threshold of 5 × 10^−6^ and pruned variants in LD with these selected variants using standard PLINK settings (*r*^2^ > 0.5, utilizing 1,000 Genomes European sample LD patterns and ± 250 Kb window) (Auton et al., 2015; Chang et al., 2015). We removed variants in an extended HLA region (chromosome 6, 25–37 Mb) due to the complex long-range LD structure in this region and because we aim to understand the function of the non-HLA genetic component of CeD. We looked for candidate genes around the clumped variants as follows. First, we considered all genomic regions around (±1 Mb) every clumped variant to also capture variants in long-range LD that are outside the standard ±500 Kb window (Yang et al., 2012). We then joined all overlapping CeD-associated regions together and looked for gene transcripts that partly or fully overlapped with the associated regions. This approach identified 58 CeD-associated regions and 1,235 candidate genes that are potentially causal for CeD.

### Gene Prioritization Using Mendelian Randomization Inverse Variance Weighting, COLOC, LD Overlap, and DEPICT

We prioritized CeD-associated genes using three eQTL-based methods—MR-IVW (Burgess and Thompson, 2017), COLOC (Giambartolomei et al., 2014), and LD overlap—and one co-regulation-based method, DEPICT (Pers et al., 2015). For the MR-IVW method, we used the independent variants identified by GCTA-COJO as instrumental variables (Yang et al., 2012; Burgess et al., 2013) to test causal relationships between changes in gene expression and CeD, as we have demonstrated that this procedure is preferred over *p*-value clumping (van der Graaf et al., 2020). MR-IVW was only performed when there were three or more independent eQTLs available (164 genes). A gene was significant for the MR-IVW test if the causal estimates passed a Bonferroni threshold *p*-value of 3.1 × 10^−4^. Heterogeneity of causal estimates was evaluated using weighted median MR analysis and Cochran's *Q*-test (Bowden et al., 2018). For the COLOC method, we used the “coloc” v3 R package and considered a gene significant for the COLOC analysis if the posterior probability of shared variants (H4) was larger than 0.9. For the LD overlap method, a gene was considered significant if there was high LD (*r*^2^ > 0.8) between the top independent eQTL and the top CeD variant in the region. Finally, we applied DEPICT (Pers et al., 2015) to the clumped CeD GWAS variants described in *Celiac Disease Summary Statistics Associated Regions and Candidate Genes*. Genes identified by the DEPICT analysis were considered significant if a false discovery rate (FDR) <0.05 was found with DEPICT's own permutation measure.

We scored each gene in the CeD-associated loci by considering each of the four prioritization methods. A gene was prioritized as “potentially causal” in CeD pathology when one of the four methods was significant (one line of evidence). If multiple lines of evidence were significant, the gene was prioritized more highly than when only a single line of evidence was available.

To explore how the prioritized genes affect CeD risk, we gave each gene an effect direction based on the effect direction of the top variants in the eQTL and the CeD GWAS. We assigned positive (“+”) when increased expression increases CeD risk, negative (“–”) when increased expression decreases CeD risk and “?” when it was not clear from our results how the expression affects CeD risk. To assign these directions, we used the following procedure:

1. If there was a concordant effect that was significant in the top variants of both the eQTLs and the GWASs, the direction of the concordant effect was chosen.
2. If there was a concordant effect but no significance of the SNP in one of the datasets, we could not be sure of an effect direction, and a question mark was chosen. The only exception to this was if the MR-IVW was significant, in which instance we chose the direction of the MR-IVW effect as effect direction.
3. If there was a discordant effect between the top SNPs and both were significant in both datasets, a question mark was chosen. The only exception to this was when the MR-IVW was significant, the MR-IVW effect was chosen.
4. If the effect between the eQTL top SNP and CeD top SNP was not in the same direction and only the eQTL top SNPs were genome-wide significant, the eQTL direction was chosen.
5. If the effect between the eQTL top SNP and CeD top SNP was not in the same direction and only the GWAS top SNPs were genome-wide significant, the GWAS direction was chosen.
6. Otherwise, a question mark was chosen.

### Co-regulation Clustering

The genes that have been prioritized may have some shared function in CeD pathology. To identify possible shared pathways, we performed co-regulation clustering analysis based on 1,588 normalized expression co-regulation principal components identified from RNA-seq information across multiple human tissues by Deelen et al. (2019). We performed pairwise Pearson correlation of our prioritized genes with these 1,588 principal components and derived a correlation Z score for each prioritized gene pair. We then performed hierarchical clustering of this Z score matrix using Ward distances and identified four clusters from the resulting dendrogram.

### *Trans*-eQTL and Mediation Analysis

A total of 238 autosomal genes that were not located in, but were associated with, a significant *trans*-eQTL variant (*p* < 5 × 10^−8^) in the CeD-associated regions were identified and used as potential targets for mediation by our associated genes in the CeD-associated loci (86 potential *cis* mediating genes). We first selected *trans*-eQTL genes that were co-expressed (Pearson *r* > 0.1, 197 gene combinations) with prioritized genes, then performed mediation analysis by running the *trans*-eQTL association again using the expression of the *cis*-eQTL gene as a covariate. We defined a *trans*-mediated gene if, after mediation analysis, the change (increase or decrease) in the effect size of the top *trans*-eQTL variant was significant according to the statistical test described in Freedman and Schatzkin (Freedman and Schatzkin, 1992). For this analysis, we used a Bonferroni-adjusted *p*-value of 3.0 × 10^−4^.

### Cell Type Proportion and *SH2B3* Expression Mediation Analysis

To assess if the *cis*-eQTL effect of *TRAFD1* was not a proxy for cell type composition, we performed mediation analyses in a fashion similar to the *trans*-mediation analysis above using cell proportions measured in a subset of individuals in the BIOS cohort. To ensure that there was no residual effect of *SH2B3* expression on the mediating effect of *TRAFD1*, we corrected the original *TRAFD1* expression levels for the expression levels of *SH2B3*, leaving *TRAFD1* expression independent of *SH2B3*, and reran the mediation analysis.

### Literature Review

We performed a REACTOME pathway (Chen et al., 2013) analysis to determine the potential function of the prioritized genes. This was complemented with a literature search (research and review papers) in Pubmed. For the coding and non-coding genes for which no studies were found, Genecards (www.genecards.org) and Gene Network v2.0 datasets (Deelen et al., 2019) were used, respectively. Information regarding the potential druggability of the prioritized genes was obtained from DrugBank (Wishart et al., 2018), the pharmacogenetics database (Whirl-Carrillo et al., 2012), and a previous study that cataloged the druggability of genes (Finan et al., 2017).

### THP-1 Experiments

#### Culturing of the THP-1 Cell Line

The cell line THP-1 (Sigma Aldrich, ECACC 88081201) was cultured in RPMI 1640 with L-glutamine and 25 mM 4-(2-hydroxyethyl)-1-piperazineethanesulfonic acid (HEPES, Gibco, catalog 52400-025) and supplemented with 10% fetal bovine serum (Gibco, catalog 10270) and 1% penicillin/streptomycin (Lonza, catalog DE17602E). The cells were passed twice per week at a density lower than 0.5 × 10^6^ cells/ml in a humidified incubator at 5% CO_2_, 37°C.

#### siRNA Treatment of THP-1 Culture

THP-1 cells were plated at 0.6 × 10^6^ cells/ml and transfected with 25 nM siRNA using Lipofectamine RNAimax transfection reagent (Invitrogen, catalog 13788), according to the manufacturer's protocol. Cells were treated with an siRNA to target *TRAFD1* (Qiagen catalog 1027416, sequence CCCAGCCGACCCATTAACAAT) [Knockdown (KD)], and cells treated with transfection mix without siRNA [wild type (WT)] or non-targeting control siRNA [scrambled (SCR)] (Qiagen catalog SI03650318, sequence undisclosed by company) were included as controls. All the treatments were performed in triplicate. Seventy-two hours after transfection, a small aliquot of cells was stained for Trypan Blue exclusion to determine cell viability and proliferation. The cells were stimulated with either lipopolysaccharide (LPS) (10 ng/ml) from *Escherichia coli* (Sigma catalog 026:B6) or media alone (unstimulated) for 4 h. Subsequently, the cells were centrifuged, and the cell pellets were suspended in lysis buffer and stored at −80°C until used for RNA and protein isolation.

#### qPCR of THP-1 Culture

The total RNA from THP-1 cells was extracted with the mirVana™ miRNA isolation kit (AMBION, catalog AM1561) and subsequently converted to cDNA using the RevertAid H Minus First Strand cDNA Synthesis Kit (Thermo Scientific, catalog K1631). qPCR was done using the Syber green mix (Bio-Rad, catalog 172-5124) and run in a QuantStudio 7 Flex Real-Time system (Applied Biosystems, catalog 448598). Primer sequences to determine KD levels of *TRAFD1* were 5′ GCTGTTAAAGAAGCATGAGGAGAC and 3′ TTGCCACATAGTTCCGTCCG. *Glyceraldehyde 3-phosphate dehydrogenase* (*GAPDH*) was used as endogenous qPCR control with primers 5′ ATGGGGAAGGTGAAGGTCG and 3′ GGGGTCATTGATGGCAACAATA. Relative expression values of *TRAFD1* were normalized to the endogenous control *GAPDH* and calculated using the ΔΔCT method, then given as a percentage relative to SCR expression levels.

#### Western Blot THP-1 Culture

Cell pellets from THP-1 cells were suspended on ice-cold lysis buffer [phosphate buffer saline (PBS) containing 2% sodium dodecyl sulfate (SDS) and complete protease inhibitor cocktail (Roche, catalog 11697498001)]. Protein concentration of cell extracts was determined using the bicinchoninic acid (BCA) protein kit (Pierce, catalog 23225). Proteins were separated on 10% SDS-polyacrylamide electrophoresis gel and transferred to a nitrocellulose membrane. After 1 h of blocking with 5% fat-free milk in Tris-Tween-Buffer-Saline, the membranes were probed for 1 h at room temperature with mouse monoclonal TRAFD1 antibody 1:1,000 (Invitrogen, catalog 8E6E7) or mouse monoclonal anti-actin antibody 1:5,000 (MP Biomedicals, catalog 08691001), followed by incubation with goat anti-mouse horseradish peroxidase-conjugated secondary antibodies 1:10,000 (Jackson ImmunoResearch, catalog 115-035-003). After three 10-min washes, the bands were detected by Lumi-Light Western blot (WB) substrate (Roche, catalog 12015200001) in a Chemidoc MP imaging system (Bio-Rad) and quantified using Image Lab™ software (Bio-Rad). The band intensity of TRAFD1 was normalized to actin, and the TRAFD1 SCR control level was set as 100%.

#### Statistical Analysis for *in vitro* Experiments in THP-1

The statistical analyses of proliferation, qPCR, and WB were performed using Prism 5 software (GraphPad Software, Inc.). Results are presented as mean ± SEM from a representative experiment. Statistical differences were evaluated using a one-tailed *t*-test.

#### RNA Sequencing in THP-1 Cells

RNA from THP-1 cells was extracted with the mirVana™ miRNA isolation kit (AMBION, catalog AM1561). Prior to library preparation, extracted RNA was analyzed on the Experion Stdsend RNA analysis kit (Bio-Rad, catalog 7007105). Here, 1 μg of total RNA was used as input for library preparation using the quant seq 3′ kit (Lexogen, catalog 015.96), according to the manufacturer's protocol. Each RNA library was sequenced on the Nextseq500 (Illumina). Low-quality reads, adaptors, and poly-A tail reads were removed from FASTQ files. The QC-ed FASTQ files were then aligned to the human_g1k_v37 Ensembl Release 75 reference genome using HISAT default settings Kim D. et al., 2015) and sorted using SAMtools (Li et al., 2009). Gene-level quantification was performed by the *featurecounts* function of the RSubread R package v1.6.2 (Liao et al., 2019). A modified Ensembl version 75 gtf file mapping only to the last 5′ 500 bps per gene was used as gene-annotation database to prevent counting of reads mapping to intra-genic A-repeats. Gene-level differential expression analysis between conditions was performed using the DESeq2 R package (Anders and Huber, 2010) after removing genes with zero counts. Differentially expressed genes (DEGs) were defined as genes presenting an absolute log2 fold change (|log2 FC|) >1 and an FDR ≤0.01 across treatment (WT vs. SCR or KD unstimulated cells). To identify the genes responding to LPS stimulation, the DEGs between unstimulated samples and their respective stimulated samples were determined. Venn diagrams were used to depict the relationships among these genes. REACTOME pathway analyses were performed to identify biological processes and pathways enriched in different sets of DEGs using the Enrichr API. Enrichments were considered significant if they were below a 0.05 FDR threshold defined by the Enrichr API (Chen et al., 2013). Raw data and count matrix are available under Gene Expression Omnibus (GEO) accession number GSE146284.

### Gene Set Permutation Analysis

It can be difficult to determine if a set of genes is “on average” more or less differentially expressed due to co-expression between the genes within the set. To mitigate this, we performed a permutation test that considers the median absolute T statistic calculated by DESeq2 (Anders and Huber, 2010). This allowed us to compare the expected differential expression of an arbitrary set of genes when exposed to a non-targeting siRNA with that of the observed differential expression of a siRNA targeting *TRAFD1*. We compare the median differential expression in the WT-SCR comparison, with the observed differential expression of the same set of genes in the SCR-KD comparison. This will still incorporate the co-expression structure of the data. To do this, we randomly selected a same-sized set of genes 1,000,000 times in each relevant experiment (WT-SCR or SCR-KD) and determined the observed median absolute T statistic. We calculated a ratio of how often the permuted value is higher than the observed value. For example, the observations can be that 1% of permuted gene sets are more differentially expressed in the WT-SCR experiment, while only 0.01% of permuted gene sets are more differentially expressed in the SCR-KD experiment. Finally, we divide these values by one another (percentage SCR-KD)/(percentage WT-SCR), to calculate a fold change in differential expression. In the example given above, this indicates that the KD is 100 times (0.01/1 = 100) more differentially expressed than expected.

### Available RNA Sequencing Datasets

Four RNA-seq datasets available to us were included to study the pattern of expression of prioritized genes. A brief description of each dataset is provided below.

(i) Whole Biopsy Samples Duodenal biopsies were obtained from 11 individuals (*n* = 6 CeD patients and *n* = 5 controls) who underwent upper gastrointestinal endoscopy (previously described) (Zorro et al., 2020). Data can be found under accession number GSE146190.
(ii) Intraepithelial Cytotoxic T Lymphocytes CD8+ T cell receptor (TCR)αβ Intra-Epithelial Cytotoxic CD8+ T Lymphocytes (IE-CTLs) cell lines were isolated from intestinal biopsies and expanded, as described previously (Jabri et al., 2000). Stimulations [interleukin (IL)-15, IFNβ, or IL21] and DEG analysis were described by Zorro et al. (2020). Data can be found under accession number GSE126409.
(iii) Gluten-Specific T Cells
  Stimulations with anti-CD3 and anti-CD28 (*n* = 22 samples per condition were done in six-well-plates coated overnight with anti-CD3 (2.5 μg/ml; Biolegend, San Diego, CA, USA) and anti-CD28 (2.5 μg/ml; Biolegend, San Diego, CA, USA) or PBS (negative control) for 0, 10, 30, and 180 min. At each time point, cells were harvested for RNA isolation. RNA was extracted with the mirVana RNA isolation kit (Ambion, Life Technologies, Carlsbad, CA, USA) according to the manufacturer's instructions. The quantity and quality of RNA were determined by Bioanalyzer (Agilent Technologies, Santa Clara, CA, USA). The sequencing libraries were prepared from 1 μg of total RNA using the TruSeq Stranded Total RNA with Ribo-Zero Globin kit (Illumina, San Diego, CA, USA) according to the manufacturer's instructions. Sequencing was done with the Illumina HiSeq2500 (Illumina, San Diego, CA, USA).
  Before alignment, the reverse complement of the fastQ sequences was taken using the FASTX-Toolkit (http://hannonlab.cshl.edu/fastx_toolkit). The alignment was done using Hisat2 version 2.0.4 against the forward strand with default alignment parameters (Kim D. et al., 2015). The reference genome index was made using hisat2-build indexer and the 1,000 genomes reference genome version GRCh37 v75, with default parameters. For the samples that had paired end data, only the first mate file was used for alignment. Reads mapping to multiple positions were removed.
  The genes were quantified using HTSeq (Love et al., 2014) version 0.6.1.p1, with options -m union, -t exon, –stranded yes, and other options on default. DE effects were quantified using the R package DEseq2 (version 1.26.0) (Love et al., 2014) and filtered on having an absolute log2 fold change (|log2 FC|) of at least 1 and an FDR <0.05. Data are available under accession number GSE146441 (Bakker et al., submitted).
(iv) Caco-2 Cells
  Stimulation of the Caco-2 cell line with 60 ng/ml IFNγ (PeproTech) and DEG analysis were previously described (Zorro Manrique, 2020). Data can be found under accession number GSE146893.

## Results

### Gene Prioritization Identifies 118 Likely Causal Celiac Disease Genes

To identify genes that most likely play a role in CeD (prioritized genes), we combined our recently published genome-wide association meta-analysis (Ricaño-Ponce et al., 2020) with (1) eQTLs derived from whole-blood transcriptomes of 3,503 Dutch individuals (Zhernakova et al., 2017) and (2) a co-regulation matrix derived from expression data in multiple different tissues and 77,000 gene expression samples (Pers et al., 2015). We selected 1,258 genes that were within 1 Mb of the 58 CeD-associated non-HLA variant regions (*p* < 5 × 10^−6^) (see Methods) and prioritized the genes that are the most likely related to CeD using four different gene prioritization methods: MR-IVW (Burgess et al., 2013), COLOC (Giambartolomei et al., 2014), LD overlap, and DEPICT (Pers et al., 2015) (Supplementary Figures 1B,C, Supplementary Table 1).

The first three methods use summary statistics of eQTLs and the CeD GWAS. MR-IVW is a test that assesses the causal relationship between a gene expression and the disease using the gene eQTLs as instruments (Burgess et al., 2013; van der Graaf et al., 2020) (see Methods). MR-IVW was applied to 162 genes, for which three or more independent *cis*-eQTL variant (at *p* < 5 × 10^−6^) were identified (see Methods) (Yang et al., 2012) and shown to be consistent after heterogeneity correction (Supplementary Table 2) (see Methods). COLOC is a variant colocalization test that assesses if there is a shared causal variant in the locus using all the variants in locus a instruments (Giambartolomei et al., 2014) (see Methods). LD overlap is an annotation approach that tests if the most associated variants in a GWAS locus are in strong LD with an eQTL (see Methods). DEPICT was the fourth method we used to prioritize genes. DEPICT is a gene prioritization method based on co-regulation in expression datasets across multiple different tissues. DEPICT identifies enrichment for co-regulated genes from genes in a GWAS locus. In contrast to the other methods, DEPICT assessed the potential role of all 1,258 genes independently of the presence of an eQTL.

**Figure 1:**
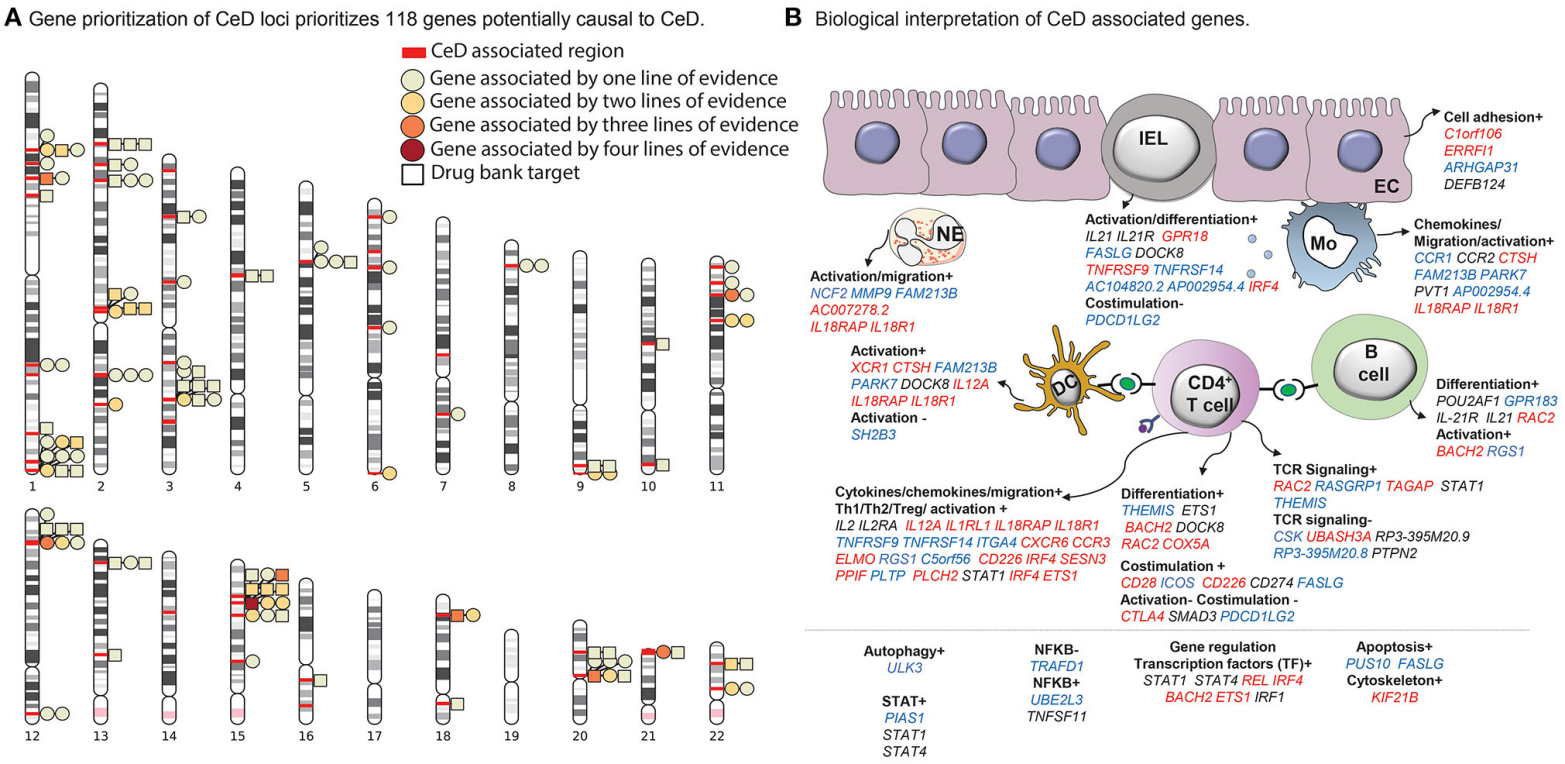
Celiac disease (CeD) prioritized genes and their proposed function and cell type. **(A)** A chromosome ideogram depicting the location of each prioritized gene identified in a CeD-associated genome-wide association study (GWAS) locus. Loci are marked with red bars. Genes depicted by a square are the target of an approved drug or a drug in development. All other genes are depicted by a circle. Each circle or square is colored according to the lines of evidence (see Methods) supporting its causal role. **(B)** Functions and cell types highlighted by the prioritized genes, according to our literature review (see Methods) (*n* = 118 genes; for 37 genes, neither a function nor a specific cell type on which the gene may operate could be specified). All genes contributing to a specific function are listed under the subheading and colored according to the change that leads to increased CeD risk: increased expression (red), decreased expression (blue), or undefined (black). The symbols + or – denote if a biological process is thought to be induced or repressed by the gene, respectively, according to literature.

In total, 118 out of the 1,258 assessed genes were prioritized by at least one of the four methods. Of these 118 genes, 28 had two lines of evidence, seven genes (*CD226, NCF2, TRAFD1, HM13, COLCA1, CTSH, UBASH3A*) had three lines of evidence, and one gene (*CSK*) was supported by all four methods (Supplementary Table 1, Figure 1A). Overall, we identified potentially causal genes in 50 out of 58 CeD-associated regions.

The four different gene prioritization methods complement each other in different ways. DEPICT prioritized the most genes: 66 in total but also had the highest proportion of 38 of them uniquely prioritized (38/66, 58% unique). One reason for this is that DEPICT is based on co-expression, not on eQTLs, and thus more genes could be tested. Indeed, 16 genes prioritized by DEPICT do not have a significant eQTL. Overall, the highest concordance between prioritization methods was found between COLOC and LD overlap (30 and 26% unique genes, respectively), while MR-IVW uniquely prioritized a relatively large proportion of genes (9/20, 45% unique). Thus, each method prioritizes genes somewhat similarly but adds a unique set of genes based on the peculiarities and assumptions of the specific method.

Interestingly, 26 of the 118 prioritized genes are targeted by an approved drug or a drug in development according to DrugBank and Finan et al. (2017) (Figure 1A, Supplementary Table 3) and therefore could lead to therapeutic interventions in CeD. For example, drugs such as natalizumab and basiliximab that target the proteins encoded by *ITGA4* and *IL21R*, respectively, are currently approved or under study for the treatment of immune-mediated diseases including rheumatoid arthritis (Chiu and Ritchlin, 2017), Crohn's disease (Rutgeerts et al., 2009), and multiple sclerosis (Baldassari and Rose, 2017) or as an immune suppressor to avoid kidney transplant rejection. An additional 25 genes encode proteins that are similar to proteins targeted by already approved drugs following Finan et al. (2017) (Supplementary Table 3).

Through a systematic literature review, we found that these 118 genes may participate in general biological processes such as cell adhesion and proliferation (*C1orf106* and *FASLG*, respectively) (Figure 1B, Supplementary Table 4) as well as immune-associated processes. For instance, genes such as *THEMIS, IL2, CD28, CTLA4*, and *UBASH3A* are involved in T cell activation and co-stimulation. Other genes including *CCR1, CC2*, and *IL21* participate in inflammation by activating and recruiting monocytes, dendritic cells, neutrophils, and B cells (Esche et al., 2005). Interestingly, we also find genes that encode for transcription factors (e.g., *IRF4* and *ETS1)* that are essential for the differentiation of T helper 1 (Th1) cells (Grenningloh et al., 2005; Mahnke et al., 2016), the key players in the pathogenesis of CeD.

### Co-regulation Patterns of *cis*-Expression Quantitative Trait Loci-Prioritized Loci Reveals Four Functional Clusters

**Figure 2:**
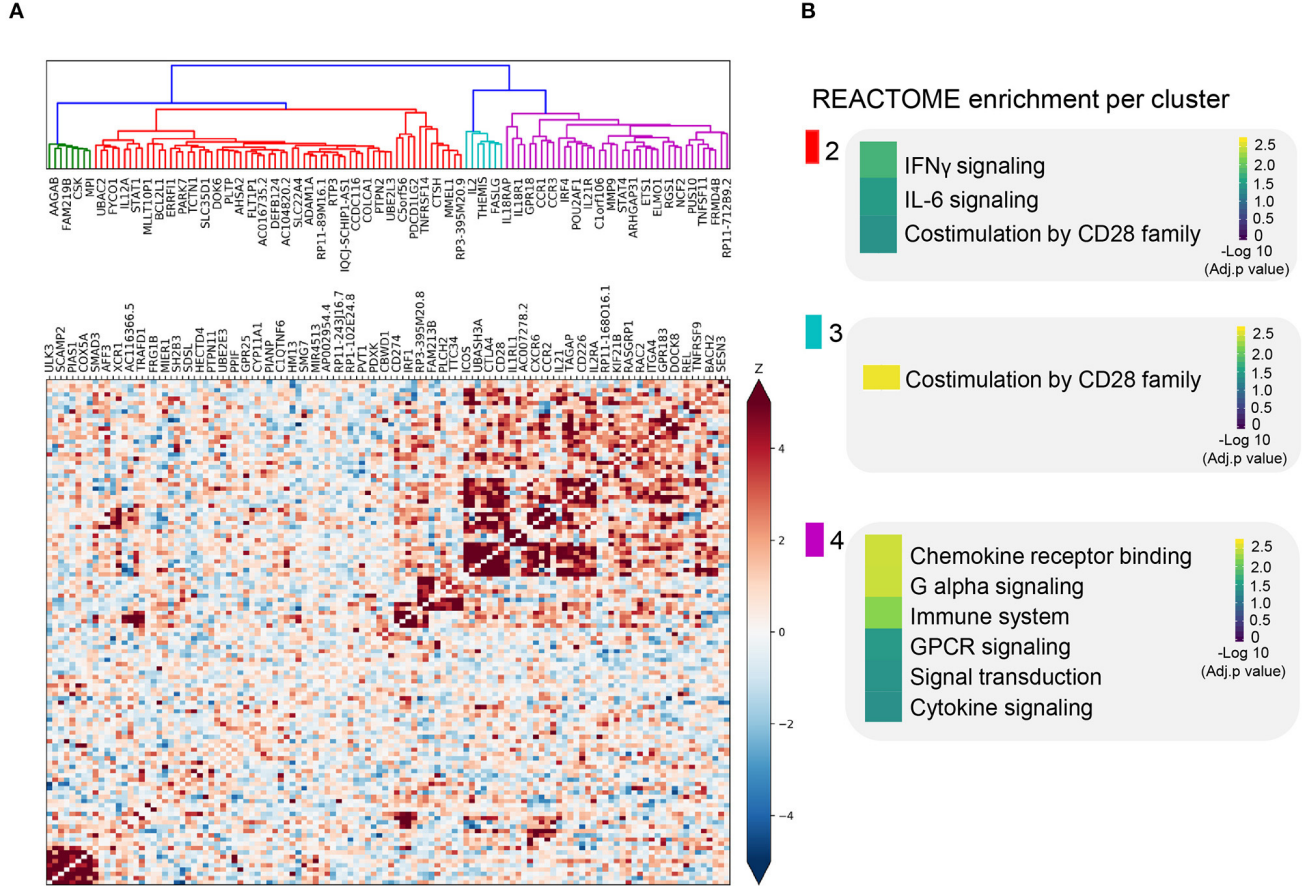
Co-expression pattern of *cis*-eQTL prioritized genes. **(A)** Heatmap showing the Spearman correlations between gene expression patterns of each prioritized gene. Blue squares indicate a negative correlation. Red squares indicate a positive correlation. Both are shaded on a gradient scale according to the Z score of the correlation. A dendrogram computed with Ward distances between the correlations is shown on top of the heatmap. Branches of the dendrogram are colored differently to mark separate clusters. **(B)** Results of the REACTOME gene set enrichment analysis of the genes belonging to each of the clusters identified in **(A)**. Color key denotes the significance (-log 10 multiple testing adjusted *p*-value) of each biological pathway.

We sought to further understand the biological role of these 118 genes using a guilt-by-association co-regulation approach to identify clusters of shared molecular function (see Methods). We identified four different clusters based on their expression co-regulation with 1,588 principal components that were identified from the co-expression of 31,499 RNA-seq samples across multiple tissues (Deelen et al., 2019) (Figure 2A, Supplementary Table 5).

In three out of four co-regulation clusters, we find shared immune function. For instance, *STAT1, CD274*, and *IL12A* are in the same cluster and are all involved in IFNγ and IL-6 signaling, later referred to as; we refer to it as the cluster enriched for IFNγ signaling (Figure 2B, Supplementary Table 6). In another cluster, we find genes involved in T cell receptor-mediated activation and CD28 co-stimulation (e.g., *CD28, CTLA4*, and *ICOS*), we refer to this cluster as the cluster enriched for TCR activation (Figure 2B). In the third cluster, we find genes involved in cytokine and chemokine signaling genes, namely, *CCR1, CCR2, CCR3, IL2EA, IL21*, and *IL18R1*; the cluster enriched for chemokine receptor binding (Figure 2B, Supplementary Table 6). The biological processes found in these clusters are essential for the activation and function of both the innate and adaptive immune systems.

The enrichments found for these clusters do not fully define the biological function of these clusters. However, this co-regulation clustering approach has the benefit that it can assign putative function to otherwise unknown genes. For example, long non-coding RNA (lncRNA) genes are usually hard to assign function to, even though they represent ~10% of our prioritized genes. Based on their cluster membership and the principle of guilt-by-association to assign function to unknown genes, most of the prioritized lncRNAs are likely to be involved in cytokine/chemokine signaling (Figures 2A,B, Supplementary Table 6).

### Mediation Analysis Uncovers *TRAFD1* as a Major *Trans*-eQTL Regulator

To further understand the potential regulatory function of the 118 prioritized genes, we identified downstream regulatory effects by performing a *trans*-mediation analysis using a two-step approach (see Methods) (Supplementary Figure 2A). We first considered all genes with a *trans*-eQTL (*p* < 5 × 10^−8^) located in any of the 58 CeD-associated regions, then performed a mediation analysis by reassessing the *trans*-eQTL effect after adjusting the expression levels for the expression of the prioritized gene(s) in the same locus.

**Figure 3:**
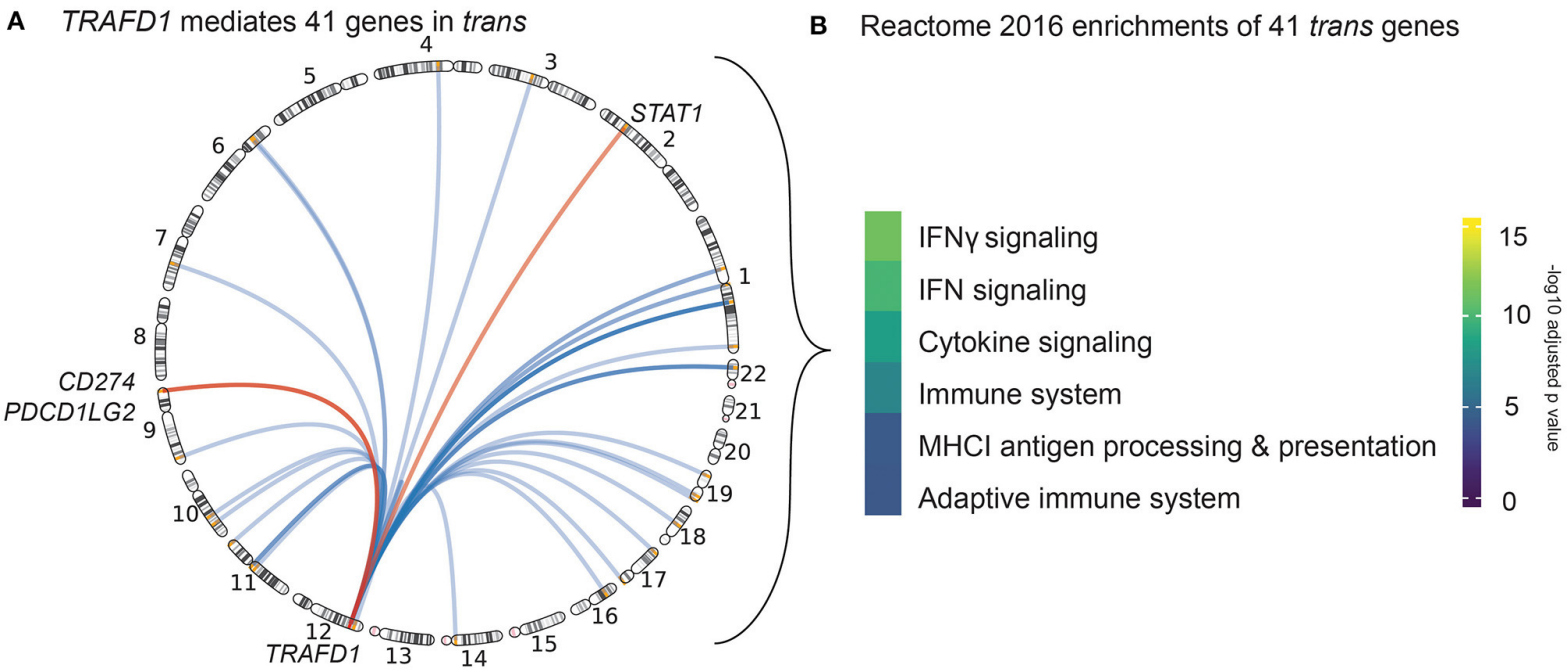
*Trans* genes action and function. **(A)** Circle genomic plot depicting the location of the 41 genes trans-mediated by *TRAFD1*. The three genes also prioritized by our *cis*-eQTL analysis are named (red). **(B)** Results of the REACTOME gene set enrichment analysis of *TRAFD1*-mediated genes. Color code denotes the significance (-log 10 adjusted *p*-value) of each biological pathway.

Of the 497 possible prioritized gene–*trans*-eQTL gene combinations, we found 172 that exhibited significant mediation effects. These combinations map to 13 associated regions and represent 21 unique mediating *cis*-eQTL genes and 79 unique mediated *trans-eQTL* genes (Supplementary Table 7). Among all the associated regions, the CeD-associated region on chromosome 12 contained the largest number of both *cis*-mediating genes (*N* = 5) and *trans*-mediated genes (*N* = 60). In this region, *TRAFD1* (which had three lines of evidence in our prioritization analysis described in *Gene Prioritization Identifies 118 Likely Causal Celiac Disease Genes*) mediated more *trans* genes (*N* = 41) than all of the other regional *cis*-regulators and also had the highest mediation impact (average Z-score difference in effect size between mediated and unmediated analysis = 2.79) (see Methods) (Supplementary Table 7, Supplementary Figures 2B,C). Of note, the top eQTL variant of *TRAFD1* is a missense variant in the nearby gene *SH2B3*. This SNP has previously been described to be a *trans*-eQTL in blood by Westra et al. (2013). In a larger *trans*-eQTL analysis, Võsa et al. (2018) found 502 significant *trans* genes. Furthermore, this missense variant has also been associated with a number of complex traits, including blood cell types and platelets, and autoimmune diseases (Astle et al., 2016; Westra et al., 2018). However, our *TRAFD1* results are not confounded by cell type, as cell type composition did not affect the eQTL association of *TRAFD1* in our cohort (*p* > 0.044 for 24 different cell type traits) (see Methods) (Supplementary Table 8). To ensure that the mediated *trans* genes of *TRAFD1* were not also mediated by *SH2B3*, we corrected *TRAFD1* expression levels for *SH2B3* expression levels and reran the mediation analysis. Here, we found that the mediating effect of *TRAFD1* was still significant for all 41 *trans-*mediated genes and that the median Z-score difference between mediated and unmediated was higher than that of *SH2B3*, although it was slightly attenuated compared to the original *TRAFD1* signal (Supplementary Table 9, Supplementary Figure 2C). Based on these results, we conclude that *TRAFD1* mediates 41 other genes in *trans* independently of *SH2B3* expression (Figure 3A).

Strikingly, three of the *TRAFD1 trans*-mediated genes—*STAT1, CD274*, and *PDCD1LG2*—are also prioritized *cis* genes in their respective loci (Figure 3A). These results suggest that the *trans*-mediated *TRAFD1* effects may have an additional additive effect in these CeD-associated loci.

*TRAFD1* is a poorly characterized gene that has been suggested to act as a negative regulator of the NFκB pathway (Sanada et al., 2008). To further elucidate the biological processes in which the 41 *TRAFD1 trans*-mediated genes could be involved, we performed a REACTOME 2016 gene set enrichment analysis (Supplementary Table 10). Here, we found that IFNγ signaling, cytokine signaling, and major histocompatibility complex class I (MHC I) antigen processing/presentation are enriched pathways, which points to a role for *TRAFD1* and *TRAFD1 trans*-mediated genes in antigen presentation and immune response (Figure 3B). Many of the TRAFD1 *trans*-mediated genes, including *GBP1/2/4/5/6, TAP1, PSME2, PSMB10, UBE2L6*, and *FBXO6*, seem involved in the processing of antigens via phagocytosis, ubiquitination, and proteasomal processing (Meunier and Broz, 2016; Øynebråten I., 2020). However, some of the *trans*-mediated genes may also be involved in IgG-dependent class II MHC antigen presentation, including *FCGR1A*, and *TRIM21* (Lu et al., 2018). This confirms that IFNγ signaling, cytokine signaling, and antigen processing and presentation are associated with CeD through genetics, independently of the HLA locus (Kumar et al., 2015).

### siRNA Knockdown of *TRAFD1* Confirms *Trans*-Mediated Genes Are Differentially Expressed

We performed a siRNA KD experiment on *TRAFD1* to gain more insights into the biological function of this gene and to validate the *TRAFD1 trans*-mediated genes in a functional assay. We evaluated the transcriptional changes of knocking down *TRAFD1* in the monocyte-like cell line THP-1 under resting conditions (unstimulated) or in the presence of LPS, a known inducer of the NFκB pathway (Dorrington and Fraser, 2019). We selected this cell line considering the role of monocytes in modulating the epithelial barrier function and cytokine secretion in a CeD-specific context (Delbue et al., 2019), as well as for the technical ability to alter the expression of *TRAFD1* using siRNA and the quick response of monocytes to LPS (Sharif et al., 2007).

**Figure 4:**
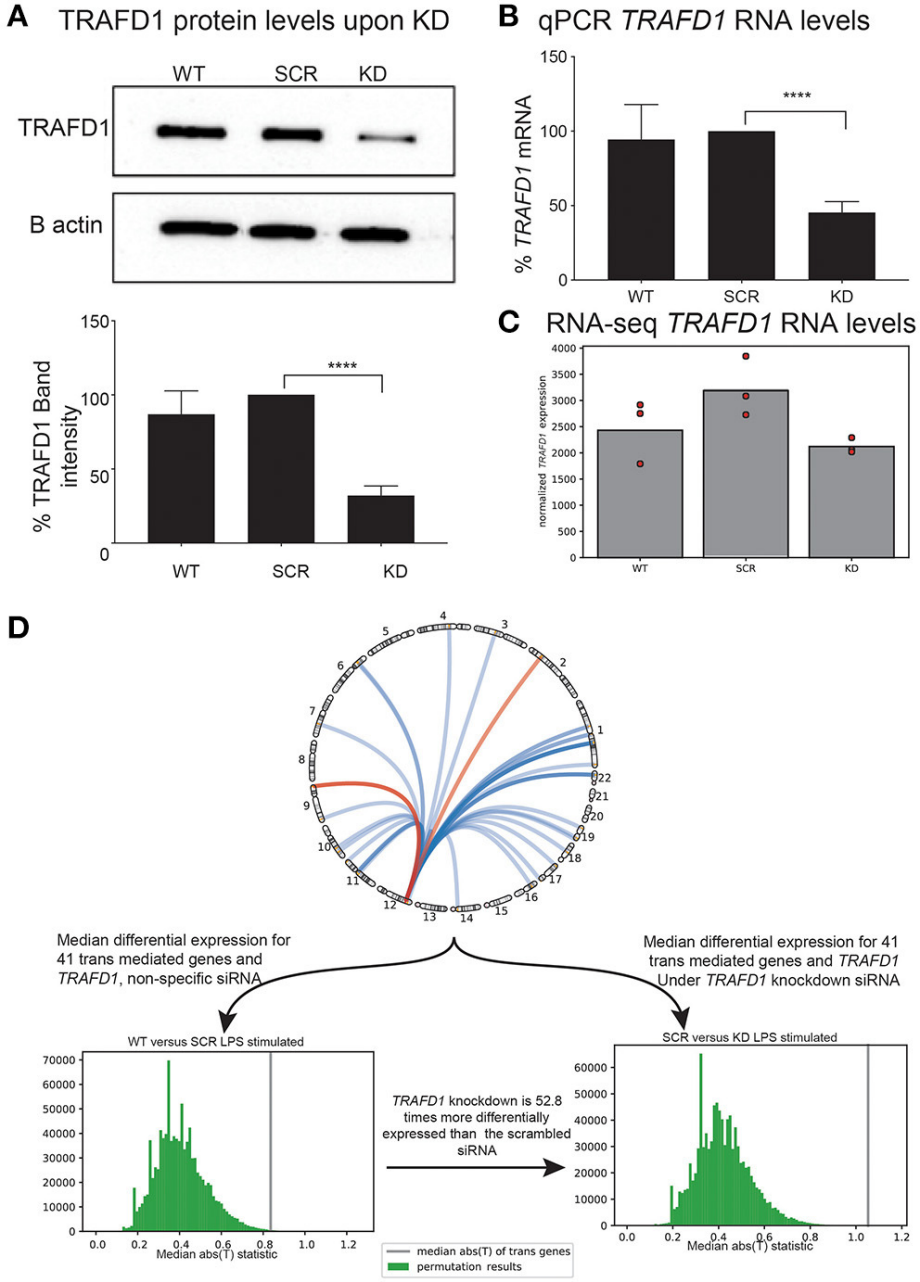
*In vitro* knockdown of *TRAFD1* validates *trans*-mediation network. THP-1 cell line based knockdown of *TRAFD1* (KD) compared to non-specific siRNA (SCR) and untransfected cells (WT) **(A)** Western blot based protein levels of *TRAFD1* compared to the B actin control. *p* ≤ 0.0001 (****). **(B)** qPCR RNA levels and **(C)** RNA-seq levels of *TRAFD1* expression. **(D)** Comparative differential expression experiment of *TRAFD1*, comparing the 41 *trans*-mediated genes and *TRAFD1* differential expression in the WT vs. SCR to the SRC vs. KD in the LPS stimulated condition. The WT vs. SCR is 52.8 times less differentially expressed as the SCR vs. KD, indicating that *TRAFD1* KD affects these 41 genes in *trans*.

After siRNA treatment, we observed no significant differences in cell viability or proliferation among the controls (WT and SCR) and the KD treatment (Supplementary Figures 3A,B). However, as expected for the KD cell line, we noted a significant reduction in the expression of *TRAFD1* compared to the controls in both WB and qPCR analyses (Figures 4A–C). KD of *TRAFD1* was also confirmed in the RNA-seq data, with *TRAFD1* expression levels reduced by 41% in unstimulated KD cells compared to unstimulated SCR cells (adjusted *p* = 0.004) and by 34% in LPS-stimulated KD cells compared to LPS-stimulated SCR cells (not significant) (Supplementary Table 11). The reduced KD effect upon LPS stimulation is consistent with our expectation that *TRAFD1* acts as a negative regulator of the NFκB pathway, which is activated by several stimuli, including LPS (Dorrington and Fraser, 2019). Therefore, we considered the KD as successful, and neither the transfection method nor a reduced expression of *TRAFD1* had a toxic effect (Figures 4A–C, Supplementary Figure 3).

Next, we tested if the 41 *TRAFD1 trans*-mediated genes were more differentially expressed than expected after LPS stimulation (Figure 4D). To disentangle differential expression from the co-expression inherently present in a gene expression dataset, we devised a permutation scheme that compared the control (WT vs. SCR) observations with the KD (SCR vs. KD) observations (see Methods). This scheme takes into account the co-expression of a gene set, as this co-expression is present in both the control and the experimental observation. After performing 1,000,000 permutations of 42 genes (41 *trans*-mediated genes and *TRAFD1*) in the LPS-stimulated comparison, the median test statistic in the control observations was observed 52.8 times more often than in the KD observations (0.264% for WT-SCR vs. 0.005% for SCR-KD, Supplementary Figure 4). This indicates that the 41 *trans*-mediated genes and *TRAFD1* as a set are 54 times more differentially expressed than expected. We did not find increased differential expression of the same gene set in the unstimulated condition (1.120% for WT-SCR vs. 0.307% for SCR-KD; Figure 4D, Supplementary Figure 4), indicating that *TRAFD1* mainly regulates genes in an LPS-stimulated state.

### RNA Sequencing in Celiac Disease-Relevant Cell Types Identifies Tissue-Specific Biological Function

To complement our REACTOME gene set enrichment analysis and dig deeper into the biological processes and cell types in which the 118 prioritized genes may act, we analyzed their expression profiles in available RNA-seq datasets from disease-relevant cell types including (1) small intestinal biopsies of active CeD patients and healthy controls; (2) IE-CTLs stimulated with disease-relevant cytokines IL-21, IL-15, and IFNβ; and (3) gluten-specific CD4^+^ T cells (gsCD4^+^ T cells) stimulated with anti-CD3–anti-CD28, which mimics the disease-specific response to gluten peptides (Supplementary Figure 5) (DEGs for each dataset are available in Supplementary Table 12).

We observed that the genes contained in the cluster not showing enrichment for particular biological functions and those contained in the cluster enriched for IFNγ signaling (green and red, Figure 2) identified in the analysis described in *Co-Regulation Patterns of cis-Expression Quantitative Trait Loci-Prioritized Loci Reveals Four Functional Clusters* are most expressed in CeD patient-derived small intestinal biopsies (Supplementary Figure 5A). Similarly, some *TRAFD1 trans*-mediated genes such as *STAT1, CXCL10*, and *TAP1*, which are essential for IFN response (Kim H. S. et al., 2015), chemotaxis (Majumder et al., 2012), and antigen processing (Seyffer and Tampé, 2015), respectively (Supplementary Figure 5B), were found to be upregulated mainly in the biopsy samples derived from CeD patients.

Genes contained in these two clusters were also highly expressed in stimulated IE-CTLs, which is in line with the IFNγ pathway enrichment observed (Figure 2). IFNγ is mainly produced by gsCD4^+^ T cells and IE-CTLs and is known to disrupt the integrity of the intestinal epithelial cells in CeD-associated villous atrophy (Nilsen et al., 1995; Wapenaar et al., 2004; Abadie et al., 2012). Moreover, most *TRAFD1 trans-*mediated genes exhibit an increase in expression in response to IFNγ in intestinal epithelial cells (Caco-2) or IFNβ in IE-CTLs (Supplementary Figure 5B).

The genes in the co-regulation clusters enriched for TCR signaling and chemokine and cytokine signaling (cyan and pink clusters, Figure 2) were instead highly expressed in gsCD4^+^T cells, especially after stimulation with anti-CD3/anti-CD28, indicating that these prioritized genes may be biologically relevant in the immediate T cell receptor response to gluten ingestion (Supplementary Figure 5A). In contrast, antiCD3–antiCD28 stimulation in gsCD4^+^T cells resulted in both upregulation and downregulation of the *TRAFD1 trans*-mediated genes, implying that *TRAFD1 trans*-mediated genes respond more strongly to IFN signaling (IFNγ or IFNβ) than to TCR activation by anti-CD3/anti-CD28 (Supplementary Figure 5B).

Taken together, the gene expression patterns of the 118 prioritized genes, when combined with information from our literature search (Figure 1B, Supplementary Tables 4, 12), suggest that these genes may control general biological processes (e.g., apoptosis, gene regulation, and cytoskeleton remodeling) as well as specific immune functions (e.g., cell adhesion, cell differentiation, and TCR signaling) in diverse cell types (e.g., T cells, neutrophils, B cells, monocytes, epithelial cells) (Supplementary Figure 5, Supplementary Table 12). The non-HLA genetic loci associated with CeD thus seem to affect a complex network of cells and biological processes. Of note, when we analyzed the enrichment of the 41 *TRAFD1 trans*-mediated genes in significantly DEGs in CeD relevant cell types, we found that the enrichment was strongest in IE-CTLs and epithelial cells upon IFN signaling (Supplementary Table 12), suggesting that *TRAFD1* and *TRAFD1*-mediated genes modulate IFN signaling possibly *via* regulation of NFκB in the context of the CeD inflammatory environment.

## Discussion

In the present study, we aimed to identify CeD candidate genes using four *in silico* methods (MR-IVW, COLOC, LD overlap, and DEPICT) and whole blood transcriptomics data from a population-based cohort. While previous studies have used at least one of these methods (Trynka et al., 2011; Withoff et al., 2016; Fernandez-Jimenez and Bilbao, 2019; Ricaño-Ponce et al., 2020), to our knowledge, this is the first effort that integrates these four different statistical approaches. This systematic prioritization approach resulted in 118 prioritized likely causal genes, including 26 that are direct targets of an approved drug or of drugs under development for other complex diseases, including autoimmune diseases. The co-expression pattern within a large RNA-seq dataset from blood (Deelen et al., 2019) suggests that these genes are involved in cytokine signaling in innate and adaptive cells as well as in T-cell receptor activation pathways.

We also ran a *trans-*mediation analysis at CeD loci by incorporating trans-eQTLs; these have mostly been ignored by other prioritization studies that have thus missed long-distance co-regulation interactions (Brynedal et al., 2017). With this analysis, we identified one of the 118 prioritized genes, *TRAFD1*, to be *trans*-regulator of 41 genes, a set of genes showing a strong enrichment in IFNγ signaling and MHC I antigen processing/presentation pathways, which are pivotal for the disease pathogenesis. Using siRNA experiments, we confirmed a significant perturbation of these 41 genes after blocking *TRAFD1* expression, a signal that was not seen instead for the 502 genes identified by Võsa et al. (2018), trans-regulated by one of the *TRAFD1* eQTL variants (data not shown). This may indicate that our mediation analysis restricted the gene set to reflect a more defined role of *TRAFD1* in CeD.

*TRAFD1* has not been related to CeD previously (Withoff et al., 2016; Ricaño-Ponce et al., 2020). This gene is thought to be a regulator of the NFκB signaling pathway (Sanada et al., 2008), a pathway that is abnormally activated in the intestinal mucosa of CeD patients (Fernandez-jimenez et al., 2014). Our results suggest for the first time a role of *TRAFD1* in signaling response to potentially both type I and type II IFNs. IFNγ-mediated type II IFN and IFNβ-mediated type I IFN activation both involve activation of the NFκB pathway (Deb et al., 2001; Pfeffer, 2011; Thapa et al., 2011; Meyerovich et al., 2016; Mitchell et al., 2019), and *TRAFD1* and *TRAFD1*-mediated genes respond strongly to both IFNs in IE-CTLs and intestinal epithelial cells. Interestingly, the *IFNG* or *IFNB* loci are not associated with CeD directly. We therefore hypothesize that *TRAFD1* provides a genetic link to the IFN response in CeD patients.

IE-CTLs, which are the effector cells in CeD, have not thus far been directly genetically associated with the disease. However, given the activation of at least half of the 41 *trans*-mediated genes in IE-CTLs upon IFN stimulation, we propose that the IE-CTLs may also be genetically linked to the disease through the action of *TRAFD1*.

We acknowledge the limitations of our study that could have restricted our findings. For example, a drawback is the limited genome coverage of the CeD summary statistics used in this study. Immunochip only measures genotypes in regions known to be associated with immune function. Therefore, our current interpretation of CeD loci is biased toward immune-related mechanisms. Only when comprehensive whole-genome CeD association analyses will become available can this bias be removed. Another constraint is the lack of a gold standard method to select relevant genes at an associated locus. In our combined gene prioritization approach, we observed that the four different statistical methods applied to our data identified unique and jointly prioritized genes. While we believe that the genes prioritized in this study represent robustly prioritized genes for CeD, it is difficult to prove that all the prioritized genes are truly causal based on statistical methodology alone. For instance, in the MR-IVW method for causality, it is impossible to rule out pleiotropic effects, even though we do find that our estimates are not overly heterogeneous (Burgess and Thompson, 2015). Moreover, for many genes, there were not enough eQTLs to apply this causality test. Functional validation of these genes in disease context is needed to rule out false positives. In this study, we used eQTLs derived from whole blood, which is a relevant tissue in CeD, but it will not capture all causal genes across different tissues (Ongen et al., 2017). We have attempted to mitigate this bias by including DEPICT in our analyses, which uses co-expression data from many tissues regardless of eQTLs. Tissue limitations also apply to the translation of the prioritized genes to the differential expression experiments performed in the disease-relevant cell types. Genes found in blood do not directly translate to other cell types.

In conclusion, this study provides a framework for predicting candidate genes and their function using a systematic *in silico* approach that could be extended to other complex diseases. Using this approach, we not only confirmed previous association between adaptive cells (gsCD4^+^ T cells and B cells) and CeD but also unveil a link between specific genes that may contribute to the disease via innate immune cells, epithelial cells, and IE-CTLs. Finally, we identified a novel master regulator, *TRAFD1*, influencing a set of genes enriched for two major pathways of immune activation, IFNγ signaling and antigen processing, which could thus be a potential target for therapeutic interventions in CeD.

## Data Availability Statement

Summary statistics of the CeD GWAS are available from the European Genome-Phenome Archive (https://www.ebi.ac.uk/ega/studies/EGAS00001003805) under accession number EGAS00001003805. The individual-level data of the BIOS cohort is available upon request from https://www.bbmri.nl/acquisition-use-analyze/bios. All RNA-seq datasets used are available under their respective GEO accession numbers defined in the Methods section.

## Ethics Statement

Ethical approval was not provided for this study on human participants because ethical approval was done by the cohorts that provided their data to us.

## BIOS Consortium

- Bastiaan T. Heijmans: Molecular Epidemiology, Department of Biomedical Data Sciences, Leiden University Medical Center, Leiden, Netherlands
- Peter A. C. 't Hoen: Department of Human Genetics, Leiden University Medical Center, Leiden, Netherlands
- Joyce B. J. van Meurs: Department of Internal Medicine, Erasmus MC, Rotterdam, Netherlands
- Rick Jansen: Department of Psychiatry, VU University Medical Center, Amsterdam, Netherlands
- Lude Franke: Department of Genetics, University Medical Centre Groningen, University of Groningen, Groningen, Netherlands; Oncode Institute, Office Jaarbeurs Innovation Mile (JIM), Utrecht, Netherlands

## Author Contributions

AG, MMZ, SS, and IJ designed the study and analyzed and interpreted the results. AG, MMZ, UV, RA-G, IR-P, SS, and IJ performed data quality control and statistical analysis. MMZ, CL, JM, and ZB performed *in vitro* experiments. AG, FK, YK-W, LMS, S-WQ, BIOS Consortium, VK, YL, LF, SW, CW, SS, and IJ contributed data to and performed *in silico* experiments for this study. AG, MMZ, SS, and IJ wrote the manuscript with critical input from VK, YL, LF, SW, and CW. All authors read and approved the manuscript.

## Conflict of Interest

The authors declare that the research was conducted in the absence of any commercial or financial relationships that could be construed as a potential conflict of interest.

## Supplementary Material

Supplementary Figure 1 *Cis*-eQTL prioritized candidate genes in CeD loci, and workflow of this study **(A)** The workflow of this study. We have combined the CeD summary statistics with the BIOS cohort and multi tissue expression data to identify a list of 118 prioritized genes likely causal for CeD pathophysiology. We further performed a *trans***-**mediation analysis to identify the downstream targets of these genes. We performed functional follow-up of the strongest *trans-*mediating genes using an siRNA screen in the THP1 cell-line. Finally, we overlay disease relevant cell types with the prioritized and *trans*-mediated genes to elucidate their action in disease-relevant cell types and conditions. **(B)** A CeD GWAS association curve at a hypothetical GWAS locus X and the eQTL association at a potential candidate gene A. In both association plots, each dot represents a SNP plotted against the genomic position (X axis) and the strength of association (Y axis). In the GWAS association curve, the top SNP is marked in red, while other SNPs above the significance threshold (dashed line) are colored according to their LD with the top SNP. In the eQTL association curve, independent eQTLs are marked in red. **(C)** A conceptual depiction of the four statistical methods applied to link a disease locus to an eQTL locus.

Supplementary Figure 2 Trans mediation analysis workflow, and *trans* mediation in the *TRAFD1* locus on chromosome 12 **(A)** Workflow illustrating the main steps to identify *trans*-eQTL genes mediated by our *cis*-prioritized genes. First, we identified *trans-*eQTLs and *trans* genes that have a significant association (*p* < 5 × 10^−8^) in our prioritized regions. Then, for every *cis* prioritized gene in the CeD-associated region, a mediation analysis was performed to determine if the *cis* gene expression explains the *trans*-eQTL effect. **(B)** Three boxes with the eQTL association curves of *TRAFD1, SERPING1*, and *SERPING1* after mediation with *TRAFD1*. **(C)** Scatter plot indicating the absolute Z difference between unmediated and mediated *trans* associations upon mediation (y axis) by all mediating *cis* genes in the *TRAFD1* region shown on the x axis as well as when correcting *TRAFD1* expression for the expression of *SH2B3* (‘*TRAFD1 – SH2B3*’).

Supplementary Figure 3 *TRAFD1* knockdown validation. Cell viability **(A)** and proliferation **(B)** of THP-1 cells that were left untransfected (WT) or transfected with non-targeting siRNA (SCR) or siRNA targeting *TRAFD1* (KD) for 72 h. **(C)** The differential expression analysis approach.

Supplementary Figure 4 DEGs upon *TRAFD1* knockdown **(A)** Heatmap showing the pattern of gene expression of *TRAFD1* and of the 41 genes it mediates, scaled by row (see details in Methods). Expression is shown in different treatments and stimulations as indicated by colored bars on top of the heatmap. **(B–D)** Comparison of the differential expression of 42 genes found in the *trans* mediation analysis of *TRAFD1* (41 *trans*-mediated genes and *TRAFD1*) with the differential expression of 42 other randomly chosen genes. The histograms (blue) show the distribution of the median absolute T statistic of DEseq of 42 randomly chosen genes, when 1,000,000 sets of genes are randomly chosen, compared to the observed value for the 42 genes that are from the *trans*-mediation analysis (red horizontal line). We compare the results of the control experiment (WT-SCR) in **(B,D)** with the results of the knockdown experiment (SCR-KD) in **(C,E)**. The fold differences between the control experiments and the knockdown experiments show how much more than expected the 42 genes are differentially expressed in the knockdown compared to the control.

Supplementary Figure 5 Differential expression of prioritized and *trans*-mediated genes in disease relevant cell types. **(A)** Heatmap depicting the scaled expression of the 118 prioritized genes belonging to the four clusters identified in Figure 2A in three available RNA-seq datasets: intestinal biopsies from controls (CTR, *n* = 5 samples) or CeD patients (CeD, *n* = 6 samples); CD8^+^ TCRαβ intraepithelial cytotoxic lymphocytes (IE-CTLs) unstimulated or treated with IL-21, IL-15, or IFNβ for 3 h (*n* = 8 samples per condition) and gsCD4^+^ T cells unstimulated or treated with anti-CD3 and anti-CD28 (aCD3) for 3 h (*n* = 22 samples per condition). Clustering was performed using the “average” method in hclust(). **(B)** Unscaled heatmaps depicting the expression of 41 *TRAFD1 trans-*mediated genes and *TRAFD1* in RNA-seq datasets from the cell types listed above as well as Caco-2 cells untreated or stimulated with IFNγ for 3 h (*n* = 8 samples per condition). Red indicates that a gene is differentially expressed (DE), blue indicates that a gene is not differentially expressed (non-DE) (FDR < 0.01 and |log2(RPKM)> 1|). Gray (none or unstimulated), pink (IFNγ), green (IFNβ), and yellow (antiCD3/antiCD28) colors indicate the type of stimulation (treatment).

Supplementary Table 1 Prioritization of genes likely causal for celiac disease (CeD). This table contains all the genes in the prioritized CeD regions and their evidence for being causal to CeD. One gene per row is shown. Columns (in order): the human build 37 coordinates of the CeD region in which the gene is located (**region**); the gene name according to the ENSEMBL GENES 96 database (human build 37) (**gene_name**); the ENSEMBL gene identifier (**ensembl_id**); the most likely effect direction (determined as described in **Methods**) (**most_likely_direction**); number of independent eQTL variants found for the gene (**n_eqtl_effects**); the effect size (**MR_ivw_effect**) and *p*-value (**MR_ivw_p_value**) of the MR-IVW test; the summary of LD overlap (**ld_overlap_summary**), with either the top eQTL variant (“top_snp”) or an independent eQTL variant (“cojo_snp”) with the *r*^2^ linkage disequilibrium between the eQTL SNP and the CeD top variant; the coloc posterior probability of causal variants being shared (**coloc_h4**); if the gene passes DEPICTs own false discovery thresholds (**depict_fdr_pass**); and the lines of evidence that are significant compared to the lines of evidence that are available for a gene (**lines_of_evidence**). Bold fields in any of the columns indicate that the prioritization method is significant according to our thresholds.

Supplementary Table 2 Sensitivity analyses for genes selected by the IVW-MR method. In this table, genes with a significant MR-IVW effect are tested for heterogeneity using the *Q*-test statistic and the MR-weighted median results as sensitivity analysis of all significant MR results. Each row contains the following information: the human build 37 coordinates of the CeD region in which the gene is located (**region**); the gene name according to the ENSEMBL GENES 96 database (human build 37) (**gene_name**); the ENSEMBL gene identifier (**ensembl_id**); the most likely direction of the effect (determined as described in **Methods**) (**most_likely_direction**); the number of independent eQTL variants found (**n_eqtl_effects**); the effect size (**MR_ivw_effect**) and *p*-value (**MR_ivw_p_value**) of the MR-IVW test; the heterogeneity *p-*value of the MR-IVW test using Cochran's Q statistic (**MR_heterogeneity_p_value**); the weighted median effect estimate (**MR_WM_beta**) and its associated *p-*value (**MR_WM_p**); the MR effect estimate after removal of potential outliers (**MR_Q_beta**); its associated *p-*value (**MR_Q_p**); the remaining variants after outlier removal (**MR_Q_ivs**) and the heterogeneity estimate (**MR_Q_heterogeneity**).

Supplementary Table 3 Druggability information for prioritized genes. This table contains all the prioritized *cis* genes in the CeD regions that are existing drug targets according to two different databases (DrugBank v5.1.4, and Finan et al., 2017). One gene per row is shown. Columns indicate (in order): the human build 37 coordinates of the CeD region in which the gene is located (**region**); the gene name according to the ENSEMBL GENES 96 database (human build 37) (**gene_name**); the ENSEMBL gene identifier (**ensembl_id**); the most likely effect direction (determined as described in **Methods**) (**most_likely_direction**); the number of independent eQTL variants found for the gene (**n_eqtl_effects**); the effect size (**MR_ivw_effect**) and *p*-value (**MR_ivw_p_value**) of the MR-IVW test; the summary of LD overlap (**ld_overlap_summary**) with either the top eQTL variant (“top_snp”) or an independent eQTL variant (“cojo_snp”) with the *r*^2^ linkage disequilibrium between the eQTL SNP and the CeD top variant; the coloc posterior probability of causal variants being shared (**coloc_h4**); if the gene passes DEPICT's own false discovery thresholds (**depict_fdr_pass**); the lines of evidence that are significant compared to the lines of evidence that are available for a gene (**lines_of_evidence**); the druggability tier based on Finan et al. (2017), with lower tiers making it more likely that the gene is druggable (Finan et al., 2017), (**druggable_tier**), and if the gene is a drug bank drug target (**drug_bank_drug_target**). Bold fields in any of the columns indicate that the prioritization method is significant according to our thresholds.

Supplementary Table 4 Functions attributable to the prioritized genes, according to our literature review (see Methods and Figure 1B). Columns describe (in order): gene name (**gene_name**); ensemble ID (**ensembl_id**); the change that leads to increased CeD risk, i.e., increased expression (+), decreased expression (–), or undefined (?) (**direction**); attributable function based in literature (**potential_function**); and literature or web-based sources (**source_1** and **source_2**). Web-based sources include Gene cards (https://www.genecards.org/) and Genenetwork (https://www.genenetwork.nl/).

Supplementary Table 5 Cluster assignments for the prioritized genes. The 118 prioritized genes were assigned to a cluster based on a guilt-by-association co-regulation approach to find shared biological mechanisms. For each gene that was prioritized (**ensembl_id** and **gene_name**), a cluster membership is given (**cluster_membership**).

Supplementary Table 6 Significant REACTOME 2016 enrichment of *cis* prioritized genes in each co-regulation cluster. Results from the enrichr API using the gene clusters of Supplementary Table 5 as query. Columns indicate: the enrichment background (**background**); the enrichment term in the background (**term_name**); the non-corrected *p-*value of enrichment for this term (**p_value)** and *Z* score (**Z_score**); enrichr combined score (**combined_score**); *cis*-prioritized genes found in each the term (**overlapping_genes**) and the multiple testing corrected *p*-value (**adjusted_p_value**). Each tab of the excel file contain the gene set enrichment for each cluster as defined in Supplementary Table 5.

Supplementary Table 7 All the significant *trans*-mediated genes from our *cis* prioritization. Each row contains a *cis–trans* gene pair described with both the ensembl id and hgnc gene name (**cis_ensembl_id**), (**cis_gene_name**), (**trans_ensembl_id**), and (**trans_gene_name**). Mediation effect and significance are shown using the Z score of the unmediated vs. the mediated estimate (using the original unmediated standard error) (**z_score_difference**) and the mediation *p-*value of the test defined by Friedman and Schatzkin (**mediation_p**).

Supplementary Table 8 Cell type mediation analysis. We calculated to what extent cell types counted in the BIOS cohort affect the most highly associated *TRAFD1* eQTL variant. Columns show (in order): the specific cell type measurements or mediator (**mediator**); the effect size after mediation by the cell type (**mediated_beta**); the original effect size (**unmediated_beta**); difference in effect sizes between mediated and unmediated (**beta_difference**); the standard error mediation effect size (**se**); the t-statistic of the beta differences (**t_statistic**); a *p*-value of the Friedman and Schatzkin test statistic (**p_value**); the Pearson correlation coefficient between *TRAFD1* and the cell type proportion (**correlation**); and the number of observations in the BIOS cohort (**n_observations**). If a mediator has a “_Perc” suffix, the cell type counts were converted into ratios. Cell type abbreviations: Baso, Basophil count; EOS, eosinophil count; HCT, haematocrit; HGB, hemoglobin; LUC, large unstained cell count; Lymph, lymphocyte count; MCH, mean corpuscular hemoglobin; MCHC, mean corpuscular hemoglobin concentration; MCV, mean corpuscular volume; Mono, Monocyte count; MPV, mean platelet volume; Neut, Neutrophil count; PLT, platelets count; RBC, red blood cell count; RDW, red blood cell distribution width; WBC, white blood cell count.

Supplementary Table 9 Mediation results when correcting *TRAFD1* expression for the nearby *SH2B3* expression. Columns are: the ENSEMBL id (**ensemble_id**); the hgnc gene name (**gene_name**); and the mediation Z score difference (**Z_score_difference**), *p*-value (**p_value**) and Pearson correlation (**correlation**) between the *trans*-eQTL top variant and the residual of *TRAFD1* expression, after correction for *SH2B3* expression.

Supplementary Table 10 Significant REACTOME 2016 enrichment of *TRAFD1-*mediated genes. Results from the enrichr API using the mediated *TRAFD1 trans* genes as query. Columns indicate: the enrichment background (**background**); the enrichment term in the background (**term_name**); the non-corrected *p-*value of enrichment for this term (**p_value)** and *Z* score (**Z_score**); enrichr combined score (**combined_score**); *cis*-prioritized genes found in each the term (**overlapping_genes**) and the multiple-testing-corrected *p*-value (**adjusted_p_value**).

Supplementary Table 11 Differential expression results of the THP-1 experiments. This table shows differential expression analysis of the THP-1 cells with 3 h LPS treatment (LPS) or without LPS (Unstim) in wild type (WT), scrambled control siRNA (SCR) or TRAFD1 knock down conditions (KD). All conditions and treatments were performed in triplicate. Complete DESEQ2 results are shown for each possible comparison in each tab. For each gene the columns show: the ensembl id per gene (**ensembl_id**); the mean corrected expression of the gene (**baseMean**); the log2 fold change of the comparison (**log2FoldChange**); the standard error of this log2 fold change (**lfcSE**); a t-statistic of the log2foldchange (**stat**); the *p*-value (**pvalue**); and the multiple testing adjusted *p*-value (**padj**). The direction of the effect is always toward the second term in the tab name: if a log2 fold change is positive and the tab name is, for example, “WT_LPS_vs_SCR_LPS,” then the expression of the gene is increased in the SCR_LPS samples compared to the WT_LPS samples.

Supplementary Table 12 Results of DE analyses from all cell-type- and context-specific data available for this study (datasets). This table lists all results for the DE analyses (Significant DE genes are defined as padj < 0.05 and log2 fold change > |1|) and a summary report of the overlap with *TRAFD1 trans*-mediated genes (**overlap with trans genes+*TRAFD1***) and relative enrichment. The DE gene lists (padj < 0.05 and log2 fold change > |1|) for each dataset are given in individual sheets. In the sheet “enrichment,” columns upregulated and downregulated indicate if the *trans*-mediated genes are up- or downregulated under stimulated conditions compared to control conditions in each dataset. Enrichment of all the *trans*-mediated genes in the DE genes was determined using a Fisher's exact test and the enrichment *p*-value is shown in the column enrichment p-val.
