# Supplementary figures and images for: Systematic Prioritization of Candidate Genes in Disease Loci Identifies TRAFD1 as a Master Regulator of IFNγ Signaling in Celiac Disease
Source: Front Genet. 2021 Jan 25;11:562434. doi: 10.3389/fgene.2020.562434 (PMC7868554; doi:10.3389/fgene.2020.562434)

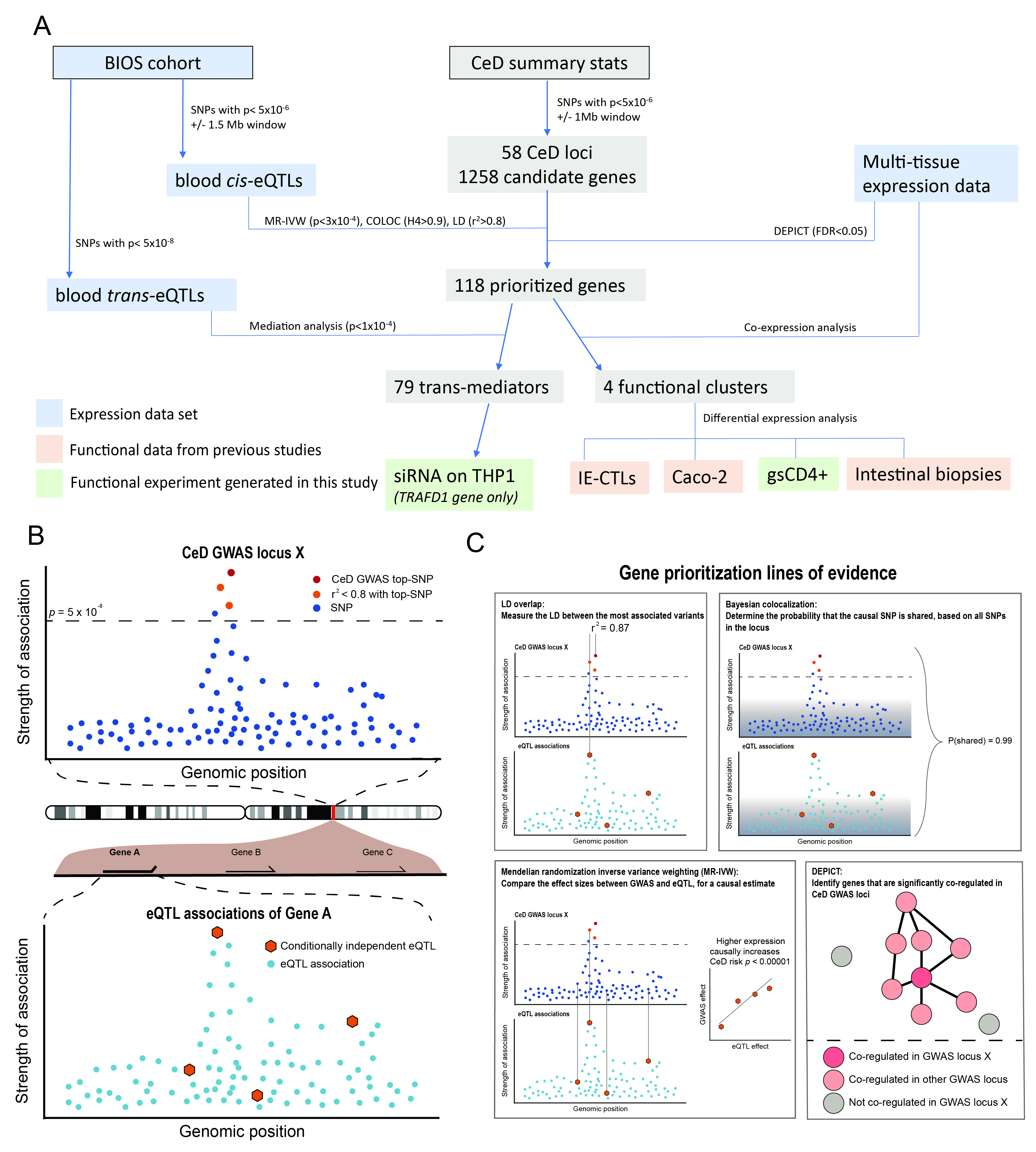

Supplement: Supplementary Figure 1 — Cis-eQTL prioritized candidate genes in CeD loci, and workflow of this study (A) The workflow of this study. We have combined the CeD summary statistics with the BIOS cohort and multi tissue expression data to identify a list of 118 prioritized genes likely causal for CeD pathophysiology. We further performed a trans-mediation analysis to identify the downstream targets of these genes. We performed functional follow-up of the strongest trans-mediating genes using an siRNA screen in the THP1 cell-line. Finally, we overlay disease relevant cell types with the prioritized and trans-mediated genes to elucidate their action in disease-relevant cell types and conditions. (B) A CeD GWAS association curve at a hypothetical GWAS locus X and the eQTL association at a potential candidate gene A. In both association plots, each dot represents a SNP plotted against the genomic position (X axis) and the strength of association (Y axis). In the GWAS association curve, the top SNP is marked in red, while other SNPs above the significance threshold (dashed line) are colored according to their LD with the top SNP. In the eQTL association curve, independent eQTLs are marked in red. (C) A conceptual depiction of the four statistical methods applied to link a disease locus to an eQTL locus. [file Image_1.JPEG]

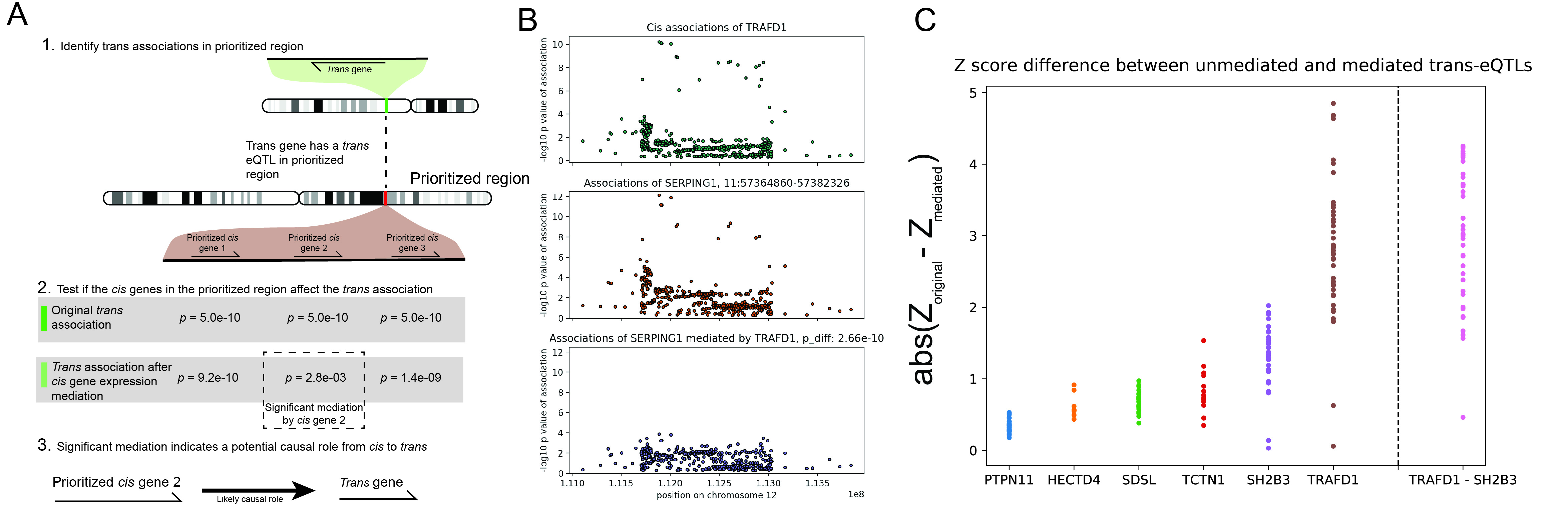

Supplement: Supplementary Figure 2 — Trans mediation analysis workflow, and trans mediation in the TRAFD1 locus on chromosome 12 (A) Workflow illustrating the main steps to identify trans-eQTL genes mediated by our cis-prioritized genes. First, we identified trans-eQTLs and trans genes that have a significant association (p < 5 × 10^−8^) in our prioritized regions. Then, for every cis prioritized gene in the CeD-associated region, a mediation analysis was performed to determine if the cis gene expression explains the trans-eQTL effect. (B) Three boxes with the eQTL association curves of TRAFD1, SERPING1, and SERPING1 after mediation with TRAFD1. (C) Scatter plot indicating the absolute Z difference between unmediated and mediated trans associations upon mediation (y axis) by all mediating cis genes in the TRAFD1 region shown on the x axis as well as when correcting TRAFD1 expression for the expression of SH2B3 (‘TRAFD1 – SH2B3’). [file Image_2.JPEG]

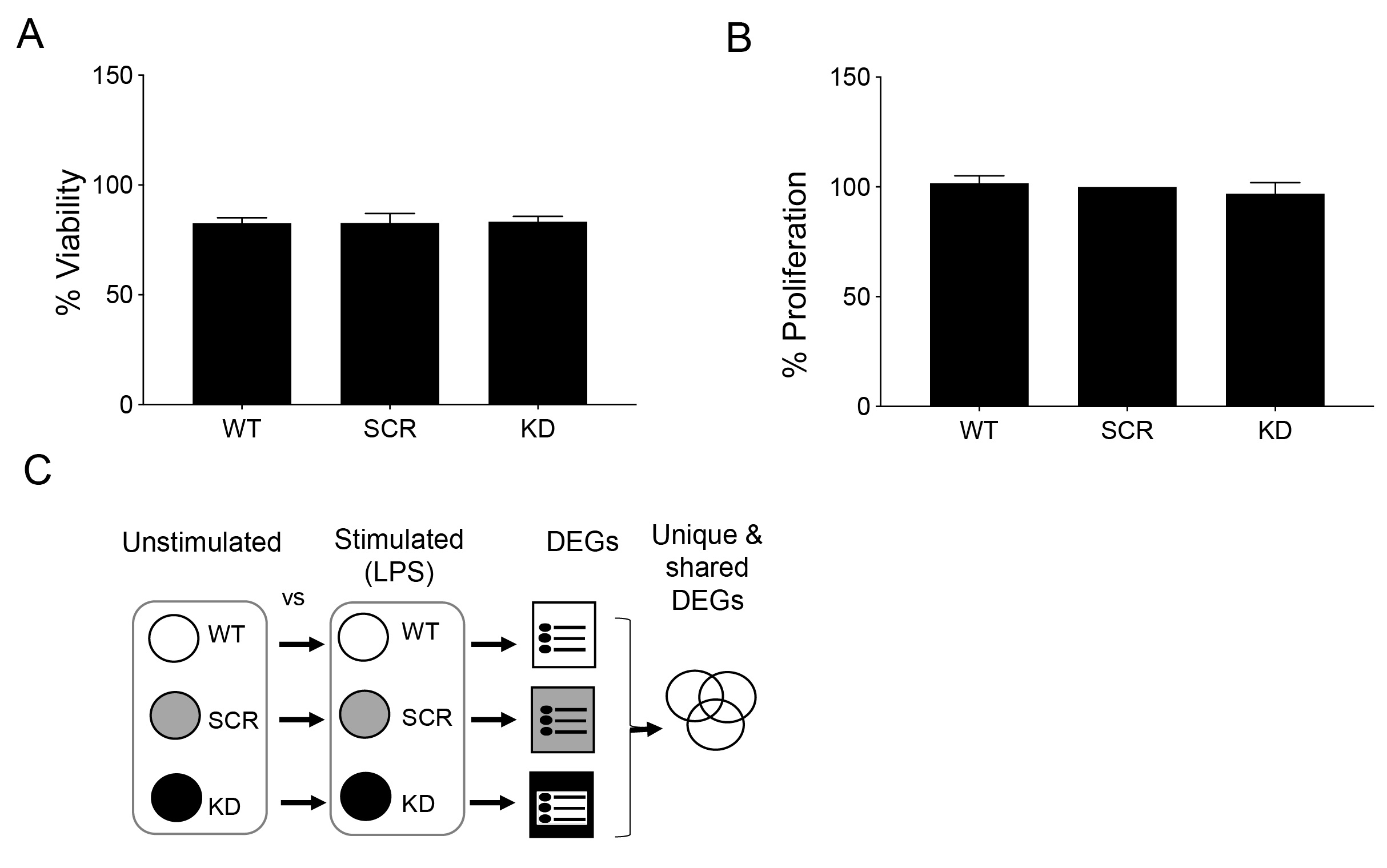

Supplement: Supplementary Figure 3 — TRAFD1 knockdown validation. Cell viability (A) and proliferation (B) of THP-1 cells that were left untransfected (WT) or transfected with non-targeting siRNA (SCR) or siRNA targeting TRAFD1 (KD) for 72 h. (C) The differential expression analysis approach. [file Image_3.JPEG]

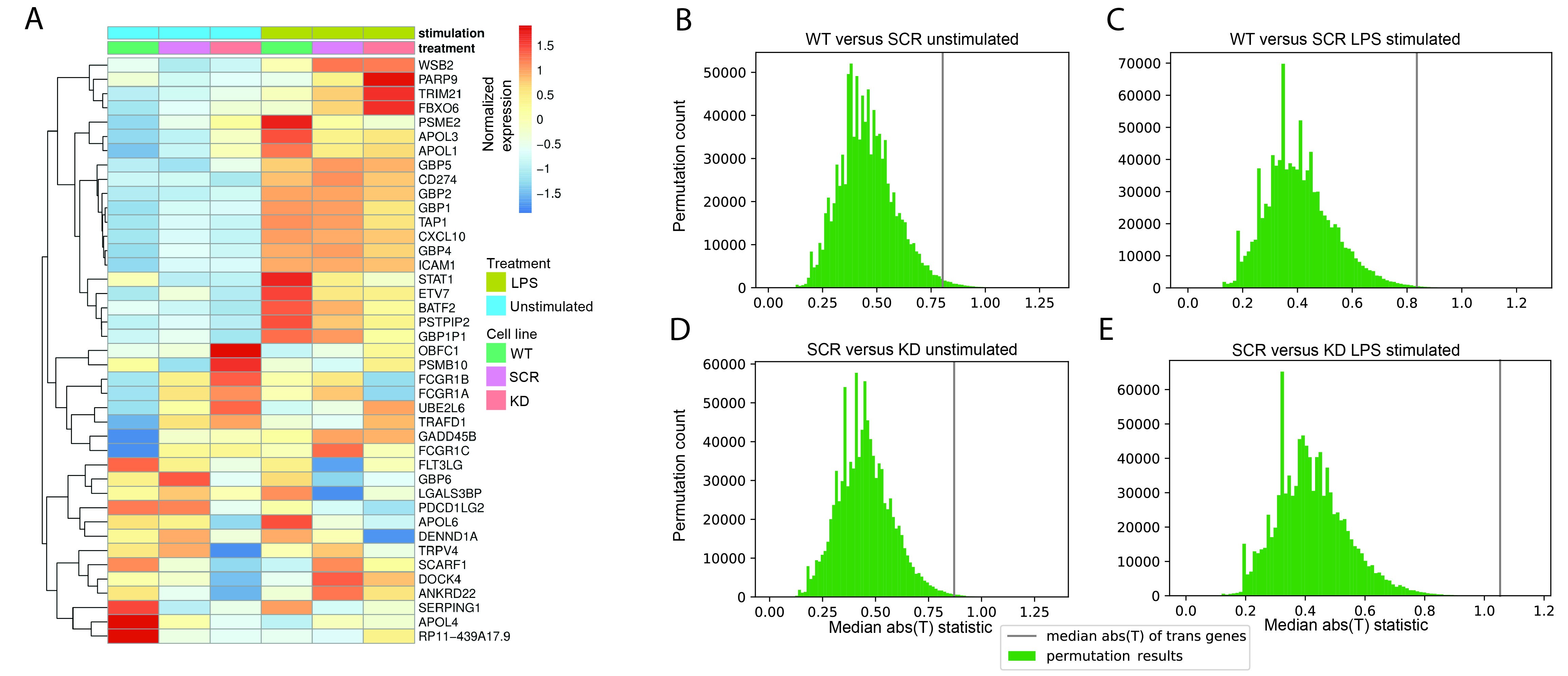

Supplement: Supplementary Figure 4 — DEGs upon TRAFD1 knockdown (A) Heatmap showing the pattern of gene expression of TRAFD1 and of the 41 genes it mediates, scaled by row (see details in Methods). Expression is shown in different treatments and stimulations as indicated by colored bars on top of the heatmap. (B–D) Comparison of the differential expression of 42 genes found in the trans mediation analysis of TRAFD1 (41 trans-mediated genes and TRAFD1) with the differential expression of 42 other randomly chosen genes. The histograms (blue) show the distribution of the median absolute T statistic of DEseq of 42 randomly chosen genes, when 1,000,000 sets of genes are randomly chosen, compared to the observed value for the 42 genes that are from the trans-mediation analysis (red horizontal line). We compare the results of the control experiment (WT-SCR) in (B,D) with the results of the knockdown experiment (SCR-KD) in (C,E). The fold differences between the control experiments and the knockdown experiments show how much more than expected the 42 genes are differentially expressed in the knockdown compared to the control. [file Image_4.JPEG]

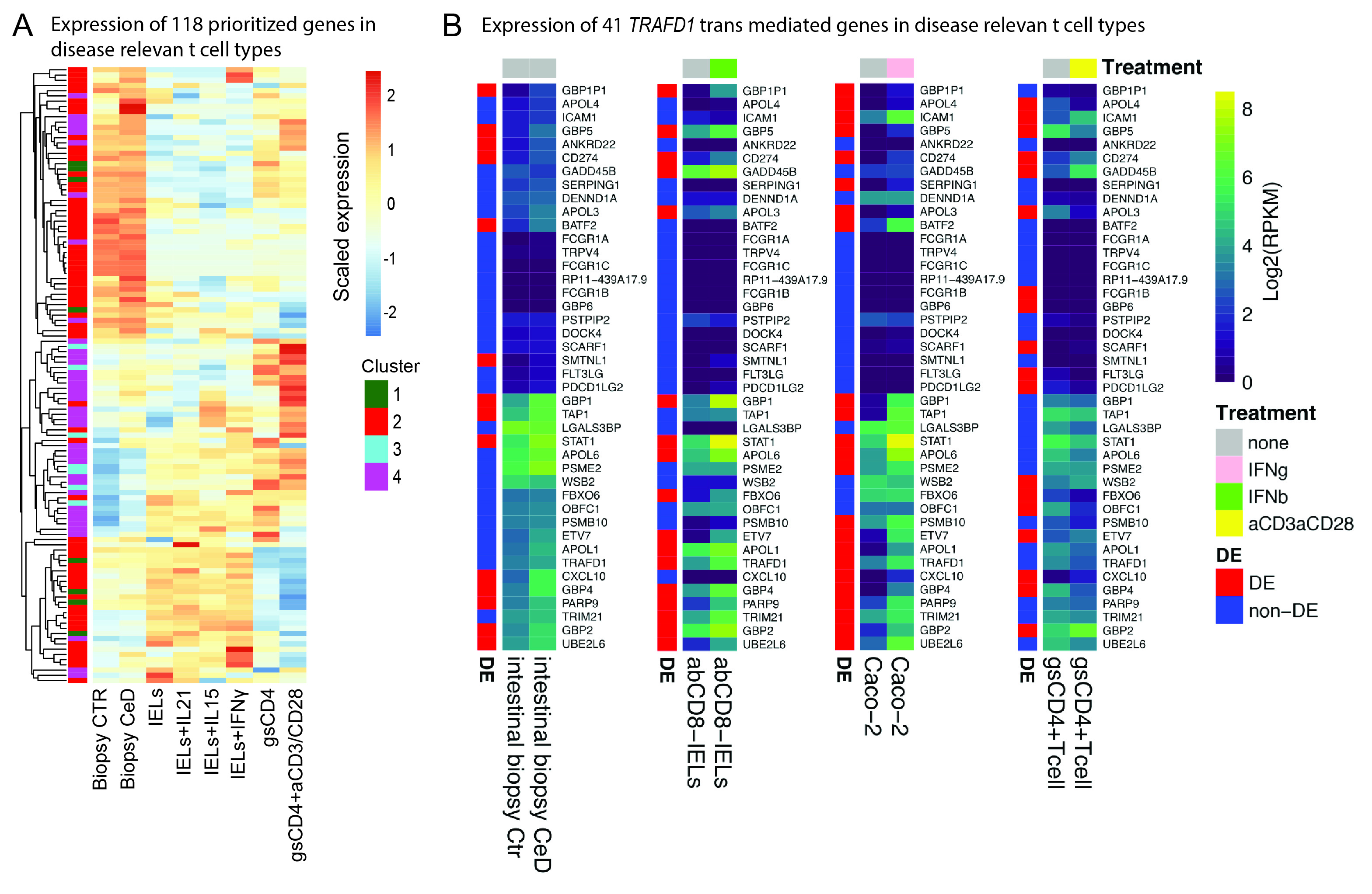

Supplement: Supplementary Figure 5 — Differential expression of prioritized and trans-mediated genes in disease relevant cell types. (A) Heatmap depicting the scaled expression of the 118 prioritized genes belonging to the four clusters identified in Figure 2A in three available RNA-seq datasets: intestinal biopsies from controls (CTR, n = 5 samples) or CeD patients (CeD, n = 6 samples); CD8+ TCRαβ intraepithelial cytotoxic lymphocytes (IE-CTLs) unstimulated or treated with IL-21, IL-15, or IFNβ for 3 h (n = 8 samples per condition) and gsCD4+ T cells unstimulated or treated with anti-CD3 and anti-CD28 (aCD3) for 3 h (n = 22 samples per condition). Clustering was performed using the “average” method in hclust(). (B) Unscaled heatmaps depicting the expression of 41 TRAFD1 trans-mediated genes and TRAFD1 in RNA-seq datasets from the cell types listed above as well as Caco-2 cells untreated or stimulated with IFNγ for 3 h (n = 8 samples per condition). Red indicates that a gene is differentially expressed (DE), blue indicates that a gene is not differentially expressed (non-DE) (FDR < 0.01 and |log2(RPKM)> 1|). Gray (none or unstimulated), pink (IFNγ), green (IFNβ), and yellow (antiCD3/antiCD28) colors indicate the type of stimulation (treatment). [file Image_5.JPEG]
